# Consensus Clustering of temporal profiles for the identification of metabolic markers of pre-diabetes in childhood (EarlyBird 73)

**DOI:** 10.1038/s41598-017-19059-2

**Published:** 2018-01-23

**Authors:** Mario Lauria, Maria Persico, Nikola Dordevic, Ornella Cominetti, Alice Matone, Joanne Hosking, Alison Jeffery, Jonathan Pinkney, Laeticia Da Silva, Corrado Priami, Ivan Montoliu, François-Pierre Martin

**Affiliations:** 1 The Microsoft Research - University of Trento Centre for Computational and Systems Biology, Piazza Manifattura 1, 38068 Rovereto, Italy; 2 Plymouth University Peninsula Schools of Medicine and Dentistry, Plymouth, UK; 3 Nestle Institute of Health Sciences, Lausanne, Switzerland; 4 Department of Mathematics, University of Trento, via Sommarive, 14, 38123 Povo, Italy; 5 Department of Computer Science, Stanford University, Stanford, CA USA

## Abstract

In longitudinal clinical studies, methodologies available for the analysis of multivariate data with multivariate methods are relatively limited. Here, we present Consensus Clustering (CClust) a new computational method based on clustering of time profiles and posterior identification of correlation between clusters and predictors. Subjects are first clustered in groups according to a response variable temporal profile, using a robust consensus-based strategy. To discover which of the remaining variables are associated with the resulting groups, a non-parametric hypothesis test is performed between groups at every time point, and then the results are aggregated according to the Fisher method. Our approach is tested through its application to the EarlyBird cohort database, which contains temporal variations of clinical, metabolic, and anthropometric profiles in a population of 150 children followed-up annually from age 5 to age 16. Our results show that our consensus-based method is able to overcome the problem of the approach-dependent results produced by current clustering algorithms, producing groups defined according to Insulin Resistance (IR) and biological age (Tanner Score). Moreover, it provides meaningful biological results confirmed by hypothesis testing with most of the main clinical variables. These results position CClust as a valid alternative for the analysis of multivariate longitudinal data.

## Introduction

More than a third of children in the UK are now overweight or obese^1^ and the increasing worldwide prevalence of obesity and type 2 diabetes (T2D) in children is a serious public health concern. It is thought that insulin resistance (IR) is an important mechanism linking obesity to the development of T2D, and recent integration of longitudinal data on IR, pubertal timing, age, sex, adiposity, and levels of the hormone Insulin-like growth factor-1(IGF-1) has highlighted a strong and gender-specific relationship between adiposity and IR in childhood^2^. Since the development of T2D can be delayed or prevented by lifestyle and medical interventions, there is increasing awareness that early identification of children with susceptibility to diabetes is critical^3^. It is important, therefore, to define the influence of childhood developmental stages on adiposity, IR and associated metabolic parameters. The EarlyBird study is a longitudinal, non-interventional cohort study of 300 healthy children in the city of Plymouth in the UK, followed annually through childhood. The study was designed to investigate the anthropometric and metabolic and endocrine processes associated with IR and prediabetes during childhood and adolescence. Metabonomic analysis was also undertaken to explore novel earlier biomarkers of adiposity and IR. In this study, we addresses the methodological challenge of integrating and correlating the temporal variations of many different data types in the EarlyBird cohort from age 5 to age 16, including anthropometric, clinical and serum metabonomic data.

In the context of longitudinal studies, methodologies have been adapted to explore the data, and to consider the multiple data dimensions, including subjects, time, and different data types. Thus, a range of solutions have been proposed for the study of longitudinal omics data, including Generalized Linear Mixed Models (GLMM), Generalized Estimating Equations (GEE), Markov models, non-parametric or semi-parametric or even Bayesian models, factor analysis, dictionary learning and latent growth curves, amongst others^4–6^. Non-parametric or semi-parametric statistical models are widely employed to model complex curves of longitudinal trajectories^7^. However, these techniques are designed to handle a single dataset generated over time. Richards *et al*. have previously summarized key approaches for intra- and inter-omic fusion strategies in a metabonomics-driven context^8^. However these integrative approaches lack the capability of accounting also for the temporal dimension. In short, a comprehensive multi-dimensional longitudinal study such as EarlyBird requires a data fusion strategy that can handle temporal profiles.

**Figure 1:**
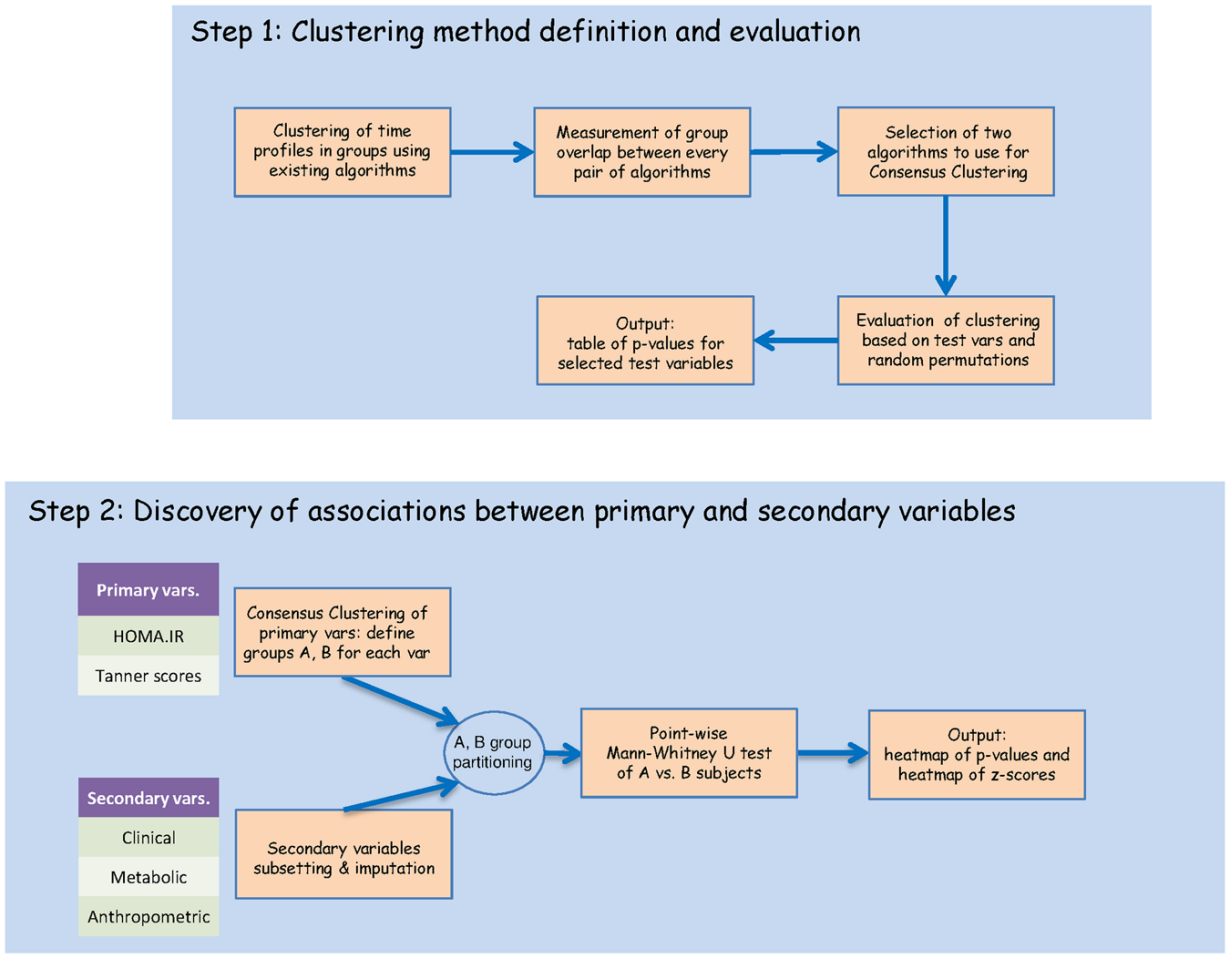
Graphical representation of the analysis workflow and indication of the variables employed at each step.

Here we report a new approach to tackle the problem of longitudinal multiple data types, and its application to the EarlyBird cohort study (Fig. 1). Briefly, we introduce the concept of primary and secondary variables, where the former are quantitative descriptors of the clinical phenotype of interest (e.g. HOMA IR), and the latter are the remaining clinical variables (anthropometric, metabolic and clinical). We use the primary variable temporal profiles to partition subjects into groups of interest (thus making effective use of the time and subject dimensions), and then to assess the relationship between these risk groups (e.g. high vs low HOMA IR) and the secondary variables.

A special challenge was represented by the partitioning of subjects based on their temporal profiles. Clustering of time series is extensively used in different areas of scientific research, and this is reflected in the abundance of different approaches that have been proposed and by the diversity of the respective sources^9^. The approaches differ in *i*) the way similarity between profiles is measured, *ii*) the algorithm that performs the partition based on the similarity matrix, and *iii*) the criterion used to decide the best number of groups (in the following we will use the terms ‘group’ and ‘cluster’ interchangeably). Given the diversity of conceptual approaches, it is not surprising that they produce different results when applied to the same dataset. In our work, the methods and packages we examined include fuzzy classification, K-means, Hierarchical clustering, Smoothing Spline Clustering (SSC), Time Series Clustering Utilities and Model-Based Clustering and Classification. As expected, we confirmed the lack of agreement of the respective procedures when run on the Earlybird dataset. As a solution to cope with these undesired effects we adopted the comparison of the output of the different methods, and then selected those producing the most robust agreement across datasets in terms of group composition. The use of the consensus between cluster compositions as the final valid clustering provides the name for our approach (Consensus Clustering).

As a final step of the workflow described here, the groups, robustly identified on the basis of a whole temporal profile comparison, are characterized in a time point-wise manner across a large panel of secondary variables. This two-step procedure simultaneously accomplishes different objectives: i) identification of the secondary variables that are biologically relevant with respect to the groups of interest (through aggregation of significance values across time points), ii) characterization of the association between risk group membership and variables found to be relevant, iii) discovery of possible age effects, and of possible co-evolution between primary and relevant secondary variables. As an example, we have assessed computational methods to perform clustering of time profiles of selected clinical variables in relation to HOMA IR trajectory and pubertal staging, and then identified correlation with other clinical, metabolic, and anthropometric data.

## Results

### Overview and description of the datasets

The EarlyBird study involved annual measurement of a range of clinical, anthropometric and metabolic variables in a cohort of children from the age of 5 to 16 years.

The *Metabolic dataset* includes repeated measurements of a panel of serum metabolites for 129 subjects. Of the original 82 species, for this work we used a subset of 46 for which an unambiguous annotation was available.

**Figure 2:**
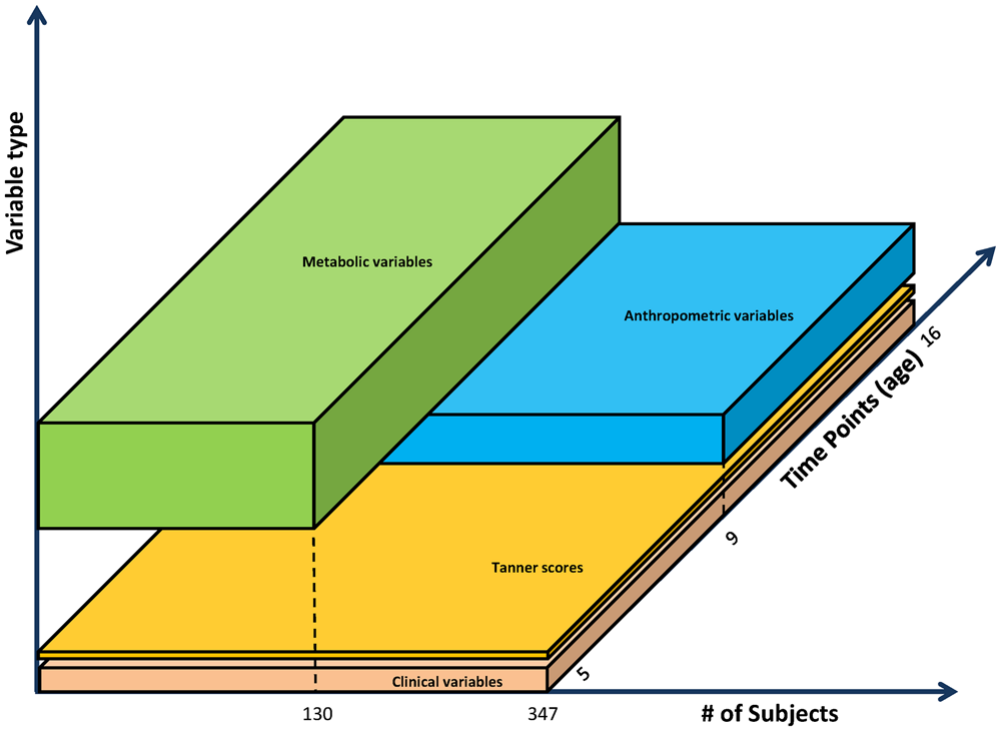
Extension of the Clinical, Anthropometric and Metabolic datasets across the subjects and temporal dimensions. The Z axis separates the individual datasets, the X axis represents number of included subjects, and the Y axis shows time points covered by each dataset.

The *Anthropometric and clinical dataset* includes repeated measurements of a panel of anthropometric and clinical variables for 149 subjects, namely body weight, body mass index, body composition data generated by dual-energy x-ray absorptiometry (DEXA), skinfold thickness, actigraphy, resting energy expenditure, and pubertal Tanner scores, fasting glucose and insulin. To deal with the gaps related to missing data when overlapping Metabolic and Anthropometric measurements, we decided to study them separately, giving us the opportunity to test our clustering method on different datasets. Figure 2 illustrates the degree of overlap between datasets: the x-axis represents measured variables, and the y-axis represents subjects; the third dimension of time is not shown for simplicity. Each dataset has a number of missing measurements: rather than imputing the missing values, we decided to restrict our analysis to subjects with complete time series. In the case of the metabolic dataset, we carried out the analysis separately for early time points and late time points, with age 11 as the dividing point. This choice was motivated by the discovery of a convergence of the metabolic parameters around age 11 in all subjects, but it also enabled the inclusion of a greater number of subjects in each analysis (since it was easier to identify subjects having complete time series over a shorter time span).

### Definition and assessment of the clustering method

We break down the complexity of the multidimensional analysis by first clustering subjects in groups based on their temporal profiles of a single clinical feature (phenotype), and then contrasting these groups with respect to each of the remaining clinical/anthropometric/metabolic profiles. Any statistically significant difference in this latter step points to clinical/anthropometric/metabolic patterns linked to the clinically determined subgroups identified in the first step.

The clustering of temporal profiles represented a challenge in itself. Several conceptual approaches have been proposed to partition a collection of temporal profiles in groups based on reciprocal similarity, which in general produce different outcomes. The approaches differ in several crucial respects: the way similarity between profiles is measured, the algorithm that performs the partition based on the computed similarities, and the criterion used to decide what should be the number of groups into which to partition the profiles. The strong dependence of the outcome on the method employed creates the problem of what clustering method and resulting partition of subjects to adopt as the starting point of the analysis. We decided to follow a strategy in which we compared the output of different methods and selected the ones that produced the most robust agreement across datasets in terms of group composition. The rationale for it is that if different conceptual approaches produce comparable outputs, then the resulting grouping is more likely to be reflecting the intrinsic properties of the profiles than those of the algorithms used. We call this strategy Consensus Clustering (CClust); we note that occasionally the same term has been used to indicate some technique to reconcile partitions resulting from different runs of the same algorithm, which is a problem unrelated to the one we are trying to solve in this work.

We adopted the availability of an implementation in the R language as a criterion for the selection of algorithms to be included in our study. This choice simplified the comparative analysis of different approaches, and enhanced the reproducibility of our workflow. More importantly, this type of selection restricts the choice of algorithms to those that have been judged worth the effort of re-implementation in a widely used language by members of the scientific community. Together, the selected methods are likely to provide a good coverage of the current state of the art in the field. The algorithms and their respective implementations we used for the comparison are fuzzy classification (package Mfuzz), K-means (R package Mfuzz), K-means (R package NbClust), Hierarchical clustering (R package NbClust), Smoothing Spline Clustering (SSC) (R package SSCLUST), Time Series Clustering Utilities (R package TSclust) and Model-Based Clustering and Classification (R package longclust).

In our consensus-based method a crucial role is played by the measure of overlap between groups produced by different algorithms. The measure we used for quantifying the degree of overlap (consistency) between each pair of algorithms was the Adjusted Rand Index, the corrected-for-chance version of the Rand index (RI). Given a set of n elements - S, and two partitions - X and Y, RI is a fraction in which the numerator is the number of agreements between partitions X and Y and the denominator is the sum of the number of agreements between partitions X and Y and the number of disagreements between partitions X and Y. While RI may only assume a value between 0 and 1, the Adjusted Rand index can assume negative values if the index is less than the expected index. Additionally, unlike the less sensitive RI, the Adjusted Rand index for two random partitionings has as an expected value, which is the constant value zero.

### Assessment of Consensus Clustering performance

Given the focus of the EarlyBird study on processes associated with IR during childhood and adolescence, HOAM IR and Tanner score were identified as variables of high interest. Moreover complete temporal series of HOMA IR and pubertal Tanner score were available for all subjects in both datasets, and so the temporal profiles of these variables were selected as primary variables.. We defined a clustering as a partitioning of subjects in n groups based on the similarity of temporal profiles; we investigated values of n equal to 2 and 3, because larger values did not provide satisfactory results in preliminary tests (not shown). We performed an assessment of the quality of the CClust by partitioning the HOMA IR and Tanner score temporal profiles and studying the separation of the average curves of the resulting groups. Since the unassisted visual inspection of the average curves was inconclusive, we devised a quantitative method in which the obtained clustering was compared with a large sample of random clustering of subjects.

#### Clustering of HOMA IR profiles: preliminary analysis

**Figure 3:**
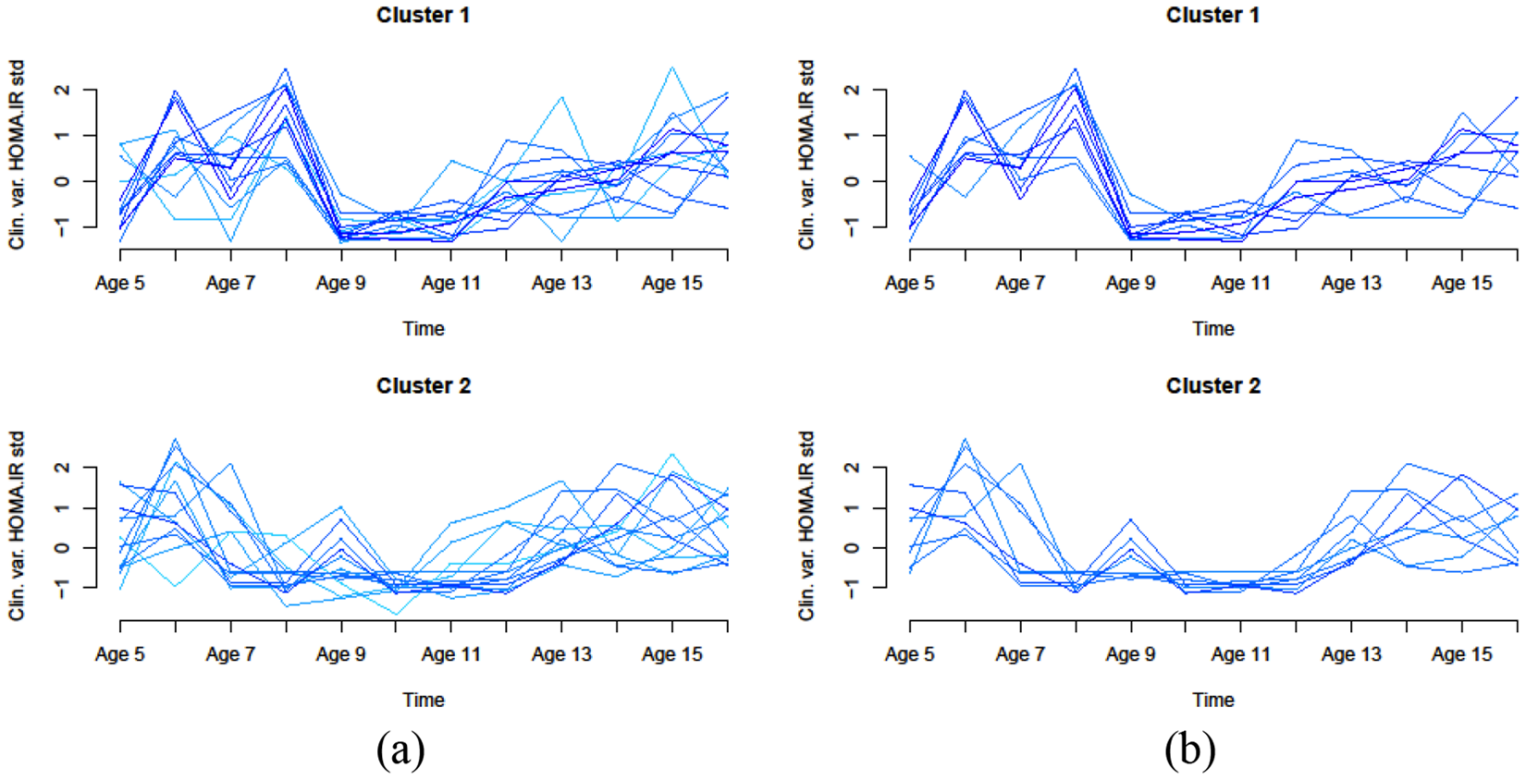
Clustering of HOMA IR complete time profiles (age 5–16) showing a convergence of the curves around age 10 (method used: soft clustering Mfuzz algorithm, number of clusters set to *n* = 2). Left panel (**a**) Resulting clusters using Mfuzz default parameter values. Right panel (**b**) curves with uncertain membership are not included in the plots (parameter min.mem set to 0.5). Other algorithm parameters: fuzzifier was set to 1.921382 (as estimated by the function mestimate) for both runs.

A preliminary analysis of the data revealed that the normalized time profiles appear to converge toward a restricted set of values around time point six (age 11), as seen in Fig. 3. While the graphs are relative to HOMA IR, the value convergence seems to represent a separation point between earlier and later metabolic phases. We took advantage of this observation, and decided to split the temporal profiles at age 11 into an early and a late segment, and to cluster the two subsets separately. The benefits of the separate clustering are a subdivision of subjects into more homogeneous groups, and the availability of larger numbers of subjects with complete time profiles over the reduced time spans.

#### Clustering of HOMA IR profiles: comparison between methods

**Figure 4:**
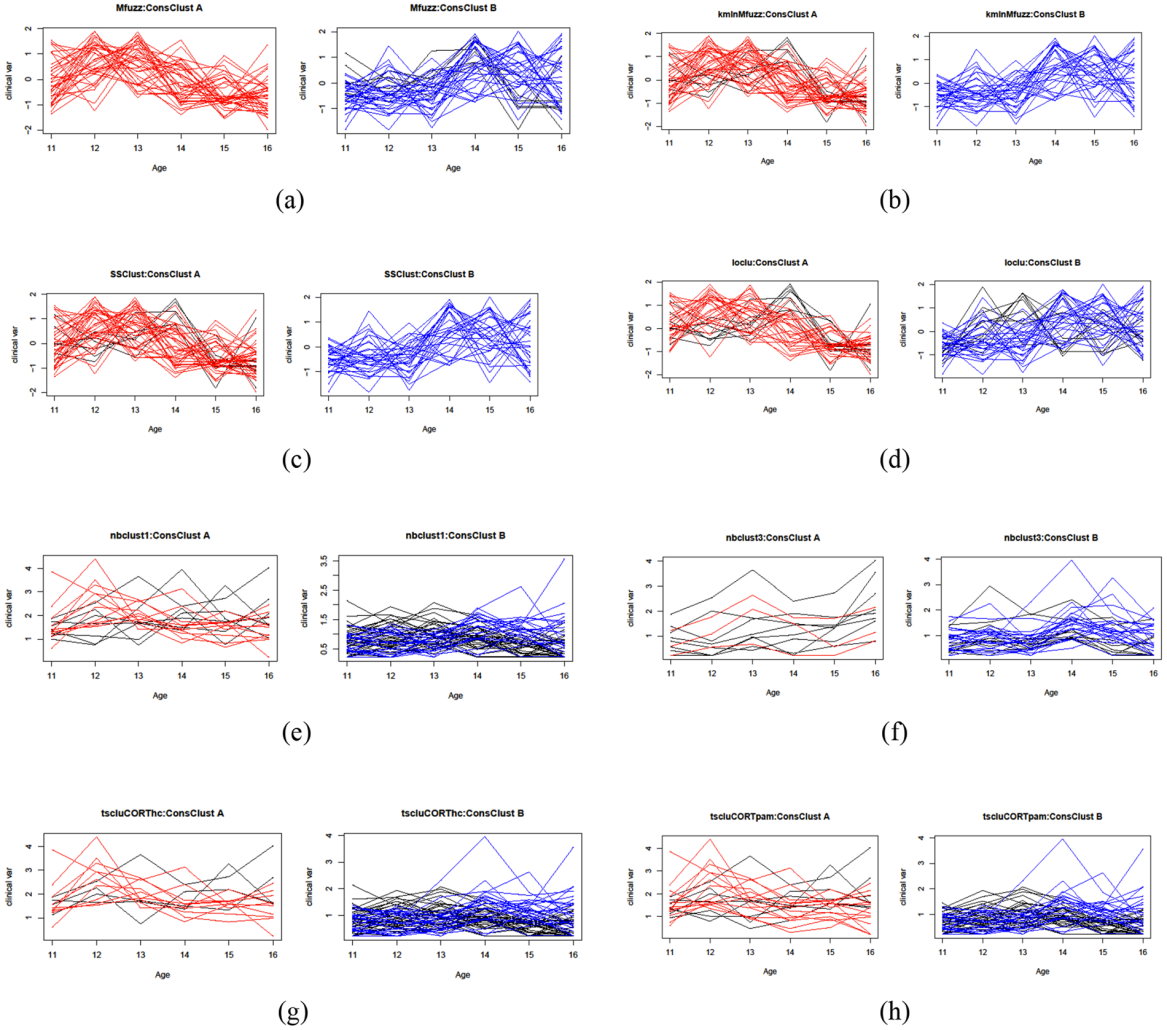
Comparison of the output generated by the different methods considered for clustering the clinical variable HOMA IR time profiles. The methods were set for n = 2 clusters, using data for both genders and age range 11–16. (**a**,**b**) Mfuzz and kmInMfuzz respectively for the fuzzy logic and the k-means algorithm (Mfuzz package); (**c**) SSClust for the Smoothing Spline Clustering method (SSC); (**d**) longclust for the model based clustering for longitudinal data; (**e**,**f**) nbclust1 and nbclust3 respectively for the clustering schemes described in the text; (**g**,**h**) tscluCORThc and tscluCORTpam respectively for the clustering schemes described in the text.

The marked difference among the results of the different clustering algorithms can be appreciated in Fig. 4. The different approaches produced inconsistent grouping of temporal profiles (i.e. having very little overlap with each other), which was not surprising given the challenging nature of the dataset (eg biological variability, age of the participants, etc…).

**Figure 5:**
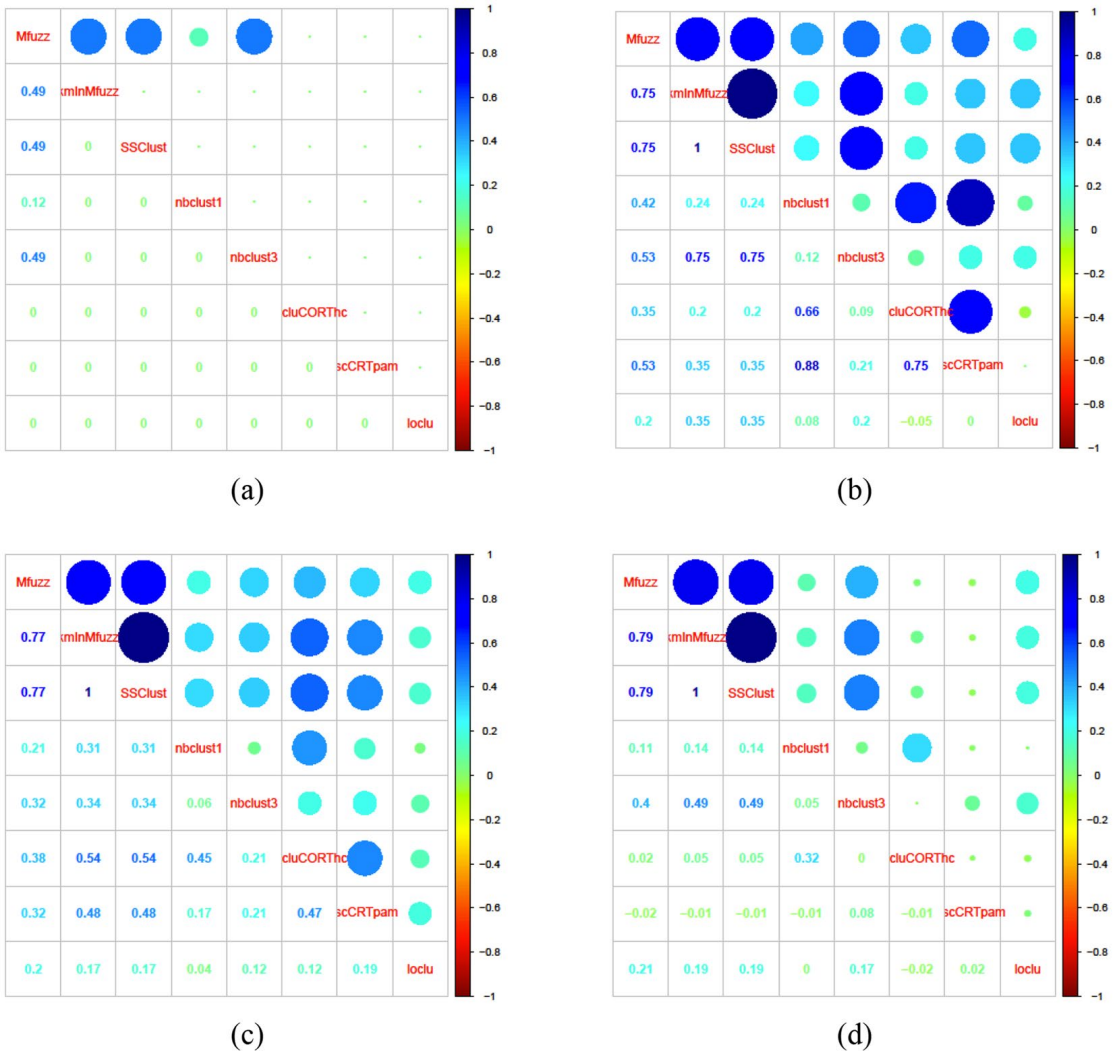
Evaluation of the overlap in composition of clusters of temporal trajectories of clinical variable HOMA IR obtained with different methods, using *n* = 2 as number of clusters. Circle color and size encode the Adjusted Rand Index for each comparison. Top row: females, (**a**) early (5–10 years) and (**b**) late time points (11–16 years). Bottom row: males (**c**) early and (**d**) late time points. The results are obtained by averaging 100 comparison between all possible pairs of clustering schemes. The following abbreviations have been used: *Mfuzz* and *kmInMfuzz* respectively for the fuzzy logic and the k-means implementations in the R package Mfuzz; nb*clust1* and *nbclust3* respectively for the clustering schemes described in the text and implemented in the R package NbClust; SS*Clust* for the Smoothing Spline Clustering method (SSC) implemented in the R package SSCLUST; ts*cluCORThc* and *tscluCORTpam* respectively for the clustering schemes described in the text and implemented in the R package TSclust.

For a systematic assessment of the level of agreement between different clustering methods we computed the Adjusted Rand Index between every pair of methods averaged across 100 runs. Figure 5 illustrates the results for the HOMA IR profiles; in this figure each panel represents a matrix in which the index is shown as a circle of size proportional to its value for every possible pair of methods (one method per row and per column). Determining an average solution over several runs was necessary because the k-means algorithm is not deterministic, and thus the partitioning of the subject can change from run to run depending on the random initial assignment of the cluster centroids. The results of the comparison suggest that Mfuzz and SSC provide a high degree of consistency across all the experiments; this result was valid for the other variables as well and not just for HOMA IR (not shown). The fact that these two methods produce highly overlapping groupings of the profiles in the first place, despite being based on very different conceptual approaches lends additional confidence in the result. Among the other methods, the pair Mfuzz/K-means showed an agreement comparable with the outcome generated by Mfuzz/SSClust, although the latter performed better on the other tested clinical variables (not shown). We therefore selected Mfuzz and SSC as the building blocks of our CClust method. As result of CClust we take the set of subjects on whose group membership the two methods agree, using a criterium of maximum overlap between the two sets of groups; the subjects on which they disagree are declared unclassifiable and discarded from the remainder of the analysis. The size of the clusters produced by CClust and used for this case study are reported in Table 1 (the groups are called A and B, where the naming is arbitrary; the size of the groups produced by the each of the two methods separately are reported in Supplementary Table 1). While we expect that the selection of the pair of methods is data-set dependent, the procedure described here is quite general and can be applied to any chosen pair.

**Table 1:** Size of A, B clusters obtained by Consensus Clustering for the HOMA IR and Tanner score time profiles.

	Group A	Group B
HOMA IR - males (Early time points)	23	9
HOMA IR - females (Early time points)	4	3
HOMA IR - males (Late time points)	31	22
HOMA IR - females (Late time points)	10	5
Tanner score - males	33	27
Tanner score - females	38	27

#### Clustering of HOMA IR profiles: permutation test

**Figure 6:**
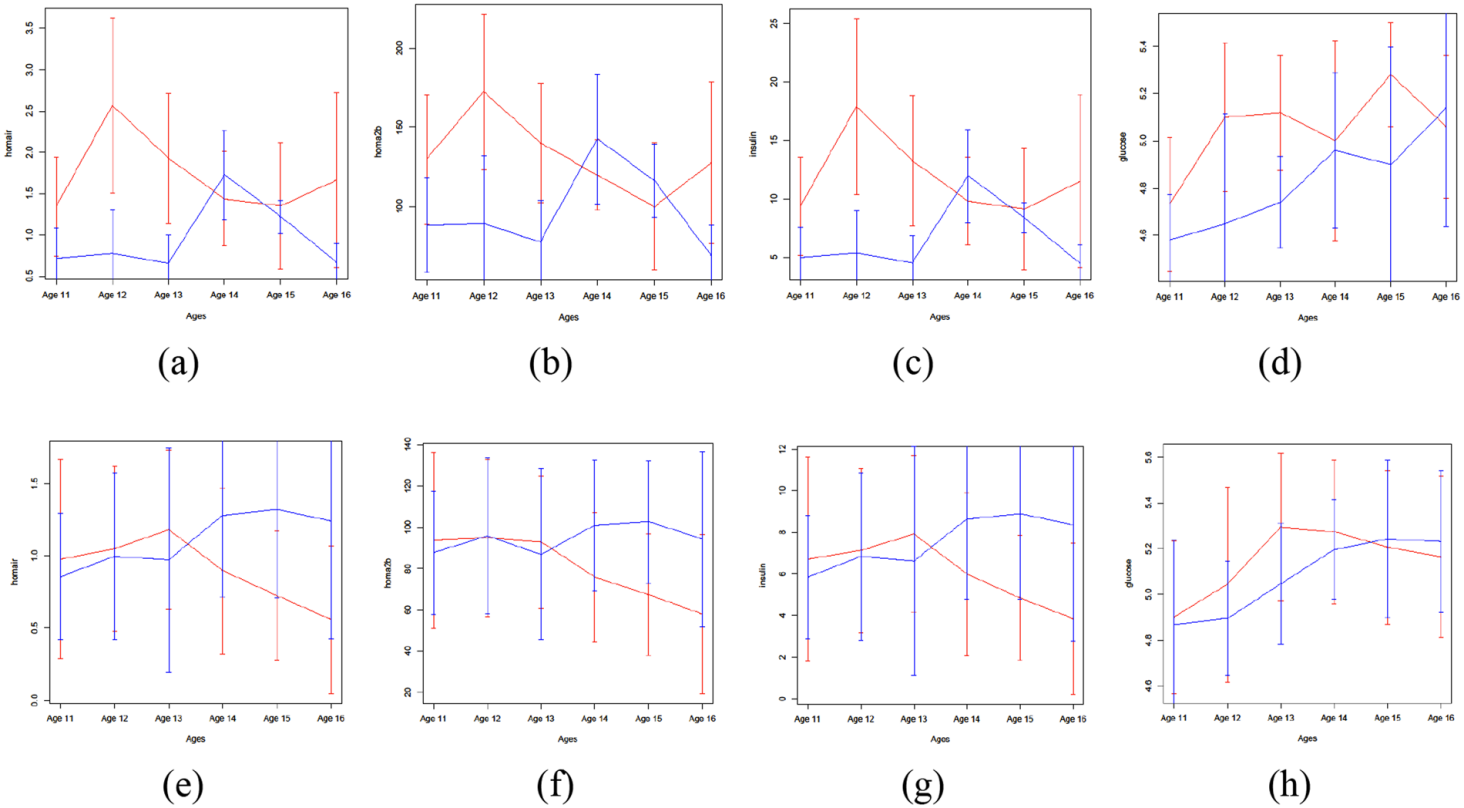
Average trajectories computed for the A, B clusters of Clinical variable HOMAIR temporal profiles (late time points). *Top:* female subjects (n = 10 and n = 5 for group A, B respectively). *Bottom:* male subjects subjects (n = 31 and n = 22 for group A, B respectively). Average curves for clinical variables HOMAIR (**a**,**e**), HOMA2B (**b**,**f**), Insulin (**c**,**g**), Glucose (**d**,**h**) with standard deviation values represented as error bars. Average curves corresponding to consensus cluster A are in red, the ones from consensus cluster B are in blue.

A first assessment of the quality of the clustering obtained as the consensus between Mfuzz and SSClust can be performed by plotting the average curves of the resulting groups. These curves, shown in Fig. 6, are the average curves of four clinical variables; group A and B averages are shown in red and blue respectively, where the two groups of subjects are those resulting from the clustering of HOMA IR curves. While the plots generally show a clear separation, the distance between curves is generally smaller than the standard deviations at each time point; therefore the result of the visual comparison is inconclusive. In order to rigorously quantify the quality of the clustering, we estimated the likelihood of observing such levels of separation by chance. We first summarized the distance between curves by adding the squares of the distance at each time point (equivalent to the square of the Euclidean distance), and we then derived an empirical distribution of values of this aggregated sum by repeatedly partitioning the subjects at random and computing the resulting sum of squares (n = 10000 permutations). The results are reported in Table 2, showing a separation significantly different from a random effect in all cases except one.

**Table 2:** Likelihood of observing a distance between average curves as large (or larger) as the one obtained by CClust over n = 10000 random partitions of the subjects in the 2-way clustering of HOMA IR temporal profiles (late time points).

	HOMA IR	HOMA.2B	Glucose	Insulin
Females	0.003	0.015	0.261	0.004
Males	0.0001	<0.0001	<0.0001	0.0001

An average curve for each of the n = 2 clusters was obtained for each one of the random partitions, and for each one of the four clinical variables shown, and the Euclidean distance between curves was computed. The values in the table indicate the percentage of random partitions producing a distance at least as large as the one measured for the CClust clusters.

### Discovery of associations between primary and secondary variables: HOMA IR-based clustering

The ultimate purpose of the clustering was to identify significant correlations between risk groups and secondary variables – metabolites, clinical and anthropometric variables. Specifically, a first question we sought to answer was whether any of the secondary variables were significantly different between groups A and B at any age. A second question of interest was whether the whole time profile of any secondary variable is significantly different between groups A and B.

#### Point-wise comparison

**Figure 7:**
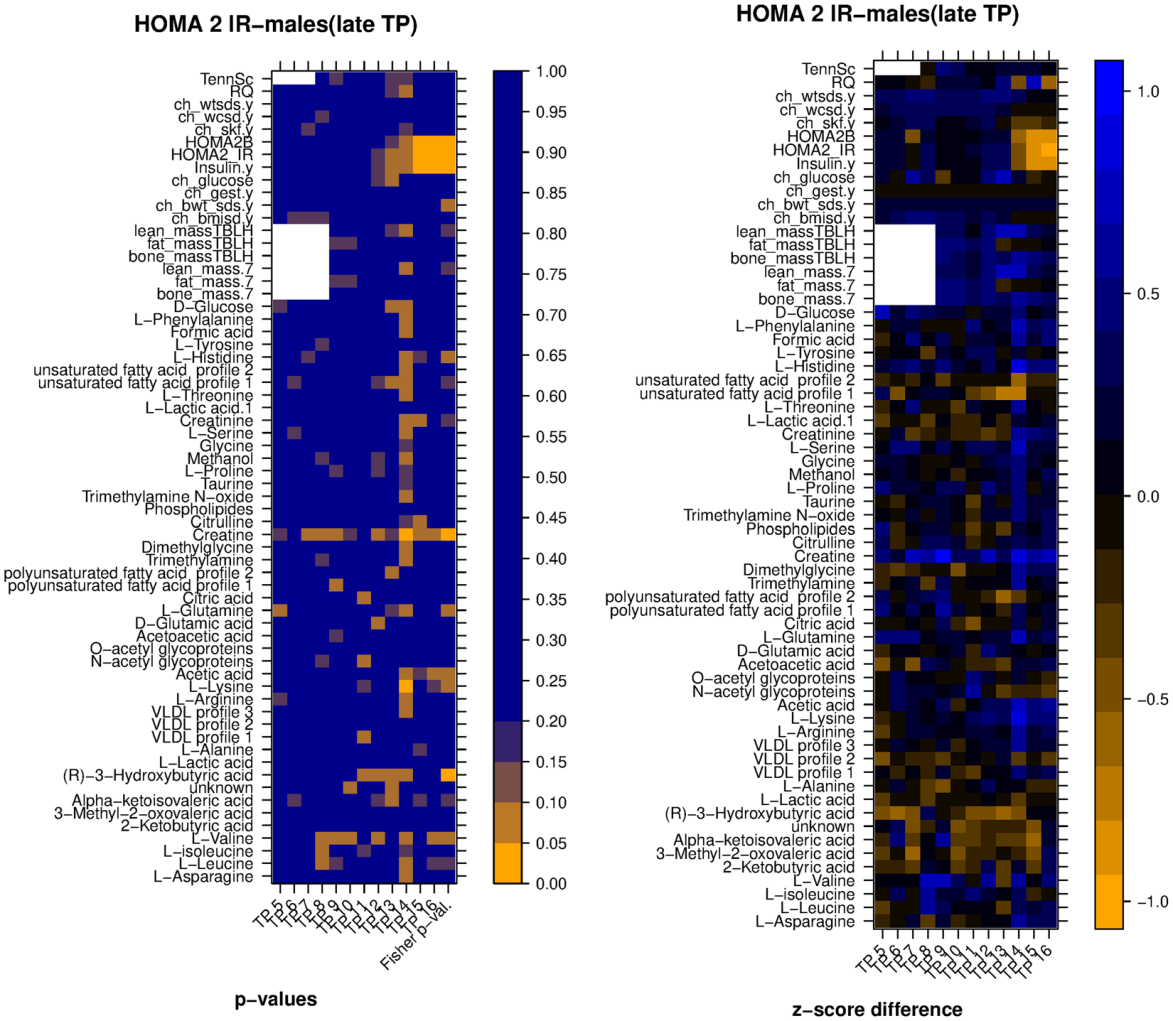
*Left:* heatmap of significance of difference between metabolic/clinic variable means between the two groups of samples (clustering according to HOMA2.IR, late time points, males only), *Right:* heatmap of difference between the normalized averages (z-scores) of the two clusters. Legend: TennSc: Tanner score, RQ; respiratory quotient; ch_wtsds, child body weight z score; ch_wcsd, child waist circumference z score; ch_glucose, child glucose; ch_gest: child gestational age; ch_bwt_sds, child birth weight z score, TBLH, total body less head.

**Figure 8:**
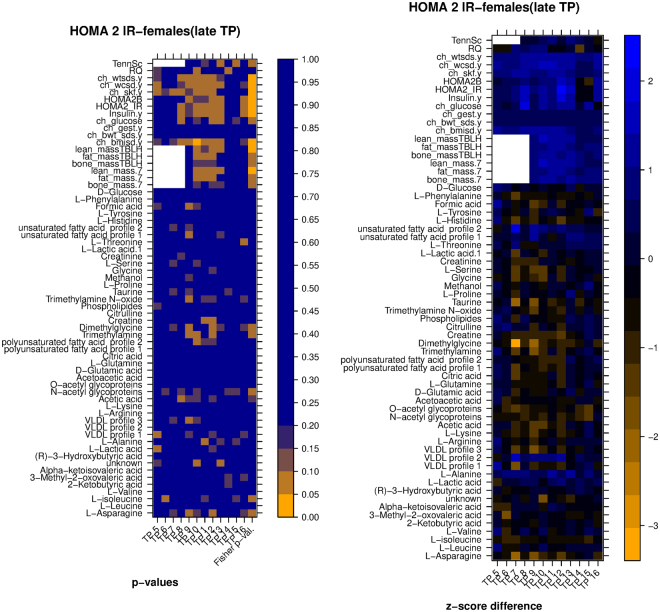
*Left:* heatmap of significance of difference between metabolic/clinic variable means between the two groups of samples (clustering according to HOMA2.IR, late time points, females only), *Right:* heatmap of difference between the normalized averages (z-scores) of the two clusters. Legend: TennSc: Tanner score, RQ; respiratory quotient; ch_wtsds, child body weight z score; ch_wcsd, child waist circumference z score; ch_glucose, child glucose; ch_gest: child gestational age; ch_bwt_sds, child birth weight z score, TBLH, total body less head.

In order to answer the first question, a comparison was performed between the values of each secondary variable for subjects in group A and group B, separately for each time point, where A and B were the groups of subjects resulting from the clustering of temporal profiles of the primary variable referred to in the figure (the assignment of the name A or B to each group is arbitrary). The results of the comparison are visualized in the form of a pair of heatmaps (Figs 7 and 8, Figures S1–S6). In each figure, the leftmost heatmap reports the results of the statistical tests, and the heatmap on the right illustrates the differences in the average values of the secondary variables.

In more detail, the values in the leftmost heatmap were computed using a nonparametric test (Matt-Whitney U test) for each of the secondary variables (one variable per row, one time point per column). A summary p-value in the last column of the heatmap was computed using Fisher’s method. For example, the left heatmap of Fig. 7 shows that two groups resulting from the clustering of HOMA IR temporal profiles of male subjects (late time points) had average Creatine values that were significantly different only at age 16 (p < 0.05), but the difference remained close to significant at other ages, with p < 0.1 for each of the two-group comparisons at ages 7, 8, 9, 12, 14, 15 and 16. Overall, the cumulative evidence across the whole age range favoring a difference between the two groups reached statistical significance as shown by the combined Fisher p-value.

The values in the rightmost heatmap were computed as follows: values for a certain variable were transformed into z-scores separately for each year (i.e. standardized to 0 mean and standard deviation of 1 considering all subjects per year), then the difference between the average z-score of group A minus the average of group B for each year was computed and represented as a color shade. A difference of 1 or −1 indicates that there was a difference of one standard deviation between the two clusters means. Thus for example the right side of Fig. 7 shows that average Creatine was higher in group A, and that the difference reached one standard deviation at ages 9 and 14.

We note that the p-values reported for this comparison were not corrected for multiple testing. None of the variables reached statistical significance when FDR correction was applied (Figure S7); this is possibly due to the limited number of subjects in each group. This, together with the exploratory nature of this work, makes us consider these p-values only as indications of significance.

#### Time profile comparison

In order to answer the second question, for each secondary variable we first summarized the time profiles of the variable separately for each of groups A and B, and then performed a A-vs-B comparison of the resulting summary profiles. We opted for a simple summarization based on the average profile, defined as the sequence of group averages at each time point. The main advantage of summarization by simple average over more sophisticated modelling approaches, such as mixed effect models, is that this makes no difficult to verify assumptions on the data (for example, in terms of presumed linearity of the time evolution), thus resulting in a broader applicability of this newly proposed method. For the subsequent comparison step, after computing an average profile of each group for each secondary variable we then measured the Euclidean distance between the average curves. To obtain a statistical significance assessment of the measured distances, for each variable we estimated how likely it was to obtain the observed distance value purely by chance, by performing a random permutation based analysis. The resulting p-values are reported in Table 3; in the table we also report the FDR-corrected p-values.

**Table 3:** Empirical p-values obtained by random permutations using HOMA IR as primary variable and metabolites as secondary ones.

Metabolite	Female p-val	Females FDR	Males p-val	Males FDR
L-asparagine	0.00775	0.07958	0.01426	0.04089
L-leucine	0.09576	0.09789	0.03131	0.04892
L-isoleucine	0.0385	0.09031	0.04877	0.05904
L-valine	0.0878	0.09393	0.00222	0.02272
2-ketobutyric acid	0.06736	0.09031	0.03037	0.04892
3-methyl-2-oxovaleric acid	0.03144	0.09031	0.03074	0.04892
Alpha-ketoisovaleric acid	0.08719	0.09393	0.012	0.03943
Unassigned metabolite signal	0.06129	0.09031	0.04616	0.05739
3-hydroxybutyric acid	0.07808	0.09031	0.00007	0.00322
L-lactic acid (^1^H signal at 1.20 ppm)	0.0562	0.09031	0.09473	0.09473
L-alanine	0.01189	0.07958	0.08229	0.08603
VLDL ^1^H signal 1	0.02936	0.09031	0.03391	0.04892
VLDL ^1^H signal 2	0.04105	0.09031	0.05111	0.05921
VLDL ^1^H signal 3	0.04694	0.09031	0.01778	0.04089
L-arginine	0.07832	0.09031	0.01047	0.03705
L-lysine	0.04167	0.09031	0.00043	0.0069
Acetic acid	0.01209	0.07958	0.00247	0.02272
N-acetyl-glycoproteins	0.01346	0.07958	0.01697	0.04089
O-acetyl-glycoproteins	0.02252	0.09031	0.05467	0.05988
Acetoacetic acid	0.07679	0.09031	0.03403	0.04892
Glutamic acid	0.09331	0.09755	0.04088	0.05429
Glutamine	0.04983	0.09031	0.0059	0.03016
Citric acid	0.06048	0.09031	0.04198	0.05429
Polyunsaturated fatty acid signal 1	0.05083	0.09031	0.07081	0.07575
Polyunsaturated fatty acid signal 2	0.01353	0.07958	0.01411	0.04089
Trimethylamine	0.03381	0.09031	0.02378	0.04591
Dimethylglycine	0.00834	0.07958	0.02318	0.04591
Creatine	0.01691	0.08643	0.00045	0.0069
Citrulline	0.053	0.09031	0.04249	0.05429
Phospholipides	0.05923	0.09031	0.03983	0.05429
Trimethylamine.N.oxide	0.01064	0.07958	0.02443	0.04591
Taurine	0.01384	0.07958	0.05264	0.05921
L-proline	0.05202	0.09031	0.02013	0.04409
Methanol	0.07076	0.09031	0.01753	0.04089
Glycine	0.0557	0.09031	0.03255	0.04892
L-serine	0.06438	0.09031	0.00799	0.03341
Creatinine	0.08476	0.09393	0.0078	0.03341
L-lactic acid (^1^H signal at 4.14 ppm)	0.1	0.1	0.09382	0.09473
L-threonine	0.03829	0.09031	0.01764	0.04089
Unsaturated fatty acid signal 1	0.03264	0.09031	0.0043	0.02826
Unsaturated fatty acid signal 2	0.05561	0.09031	0.02495	0.04591
L-histidine	0.02047	0.09031	0.0032	0.02453
L-tyrosine	0.06184	0.09031	0.05277	0.05921
Formic acid	0.07178	0.09031	0.00954	0.03657
L-phenylalanine	0.06624	0.09031	0.00527	0.03016
Glucose	0.07853	0.09031	0.03085	0.04892

Numbers shown indicate the likelihood of observing a distance between the reported metabolite average curves over n = 10000 random partitions which is as large (or larger) as the one obtained by 2-way clustering of HOMA IR profiles using our algorithm. Asterisks mark the statistically significant values (p < 0.05).

### Discovery of associations between primary and secondary variables: Tanner score-based clustering

**Figure 9:**
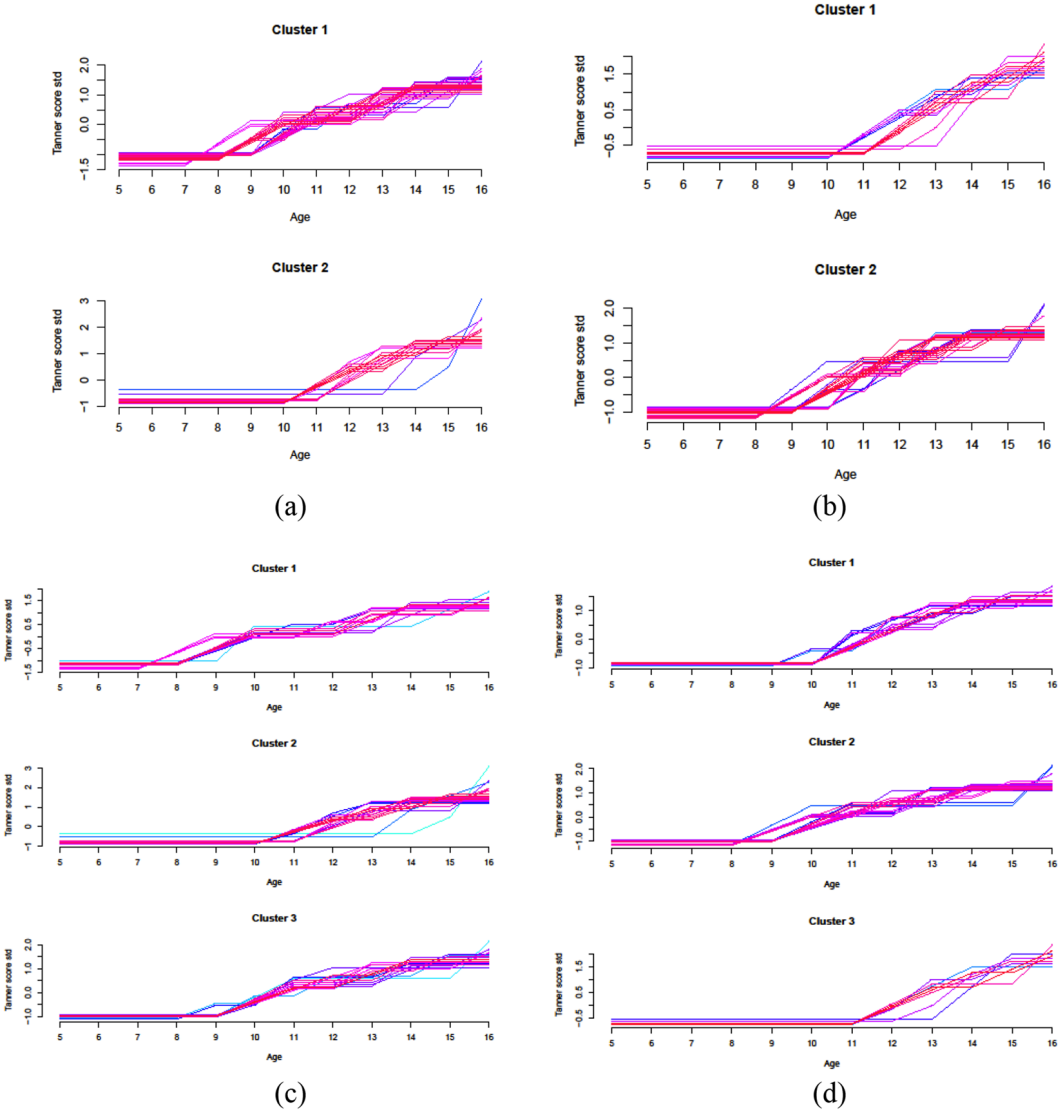
Comparison of Tanner score time profile clustering performed imposing either a 2-cluster solution or a 3-cluster solution. *(Top row)* 2-way clustering: (**a**) males, (**b**) females. *(Bottom row)* 3-way clustering: (**c**) males, (**d**) females. The clustering were obtained with Mfuzz: dark colors represent the group membership, and light blue colors show the trajectories associated to low values of membership according to the fuzzy setting of the algorithm. Mfuzz output is shown here in order to account for the trajectories of uncertain classification.

We repeated the above procedure on the dataset of 129 subjects for which we had metabolite measurements. This time we used the Tanner score temporal profiles as primary variables, and we also compared the results of the 2-way and 3-way clusterings, obtained forcing the number of desired groups to 2 and 3 respectively. Both 2-way and 3-way clustering produced well defined subject groups (Fig. 9), that could be interpreted as “early” and “late” stages in the former, and “early”, “intermediate” and “late” stage in the latter.

**Figure 10:**
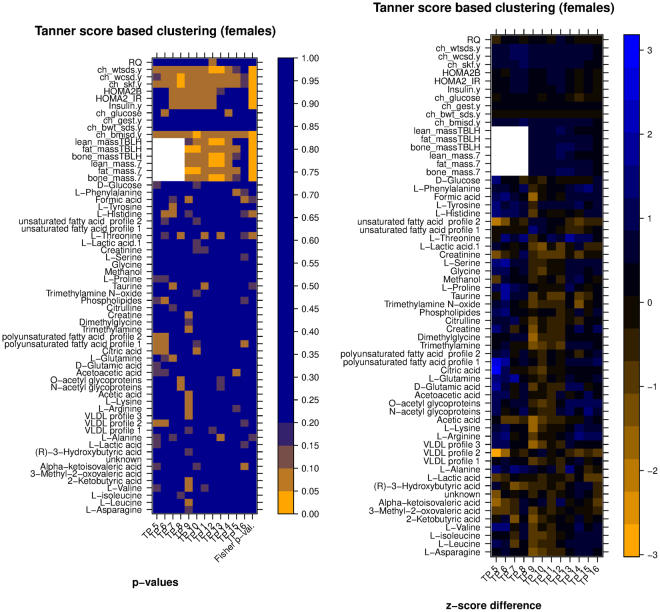
*Left:* heatmap of significance of difference between metabolic variable means between the two groups of samples (clustering by CClust according to Tanner score, all time points, females only), *Right:* heatmap of difference between the normalized averages (z-scores) of the two clusters. White boxes are fill-ins for the anthropometric variables missing time points. Legend: TennSc: Tanner score, RQ; respiratory quotient; ch_wtsds, child body weight z score; ch_wcsd, child waist circumference z score; ch_glucose, child glucose; ch_gest: child gestational age; ch_bwt_sds, child birth weight z score, TBLH, total body less head.

**Figure 11:**
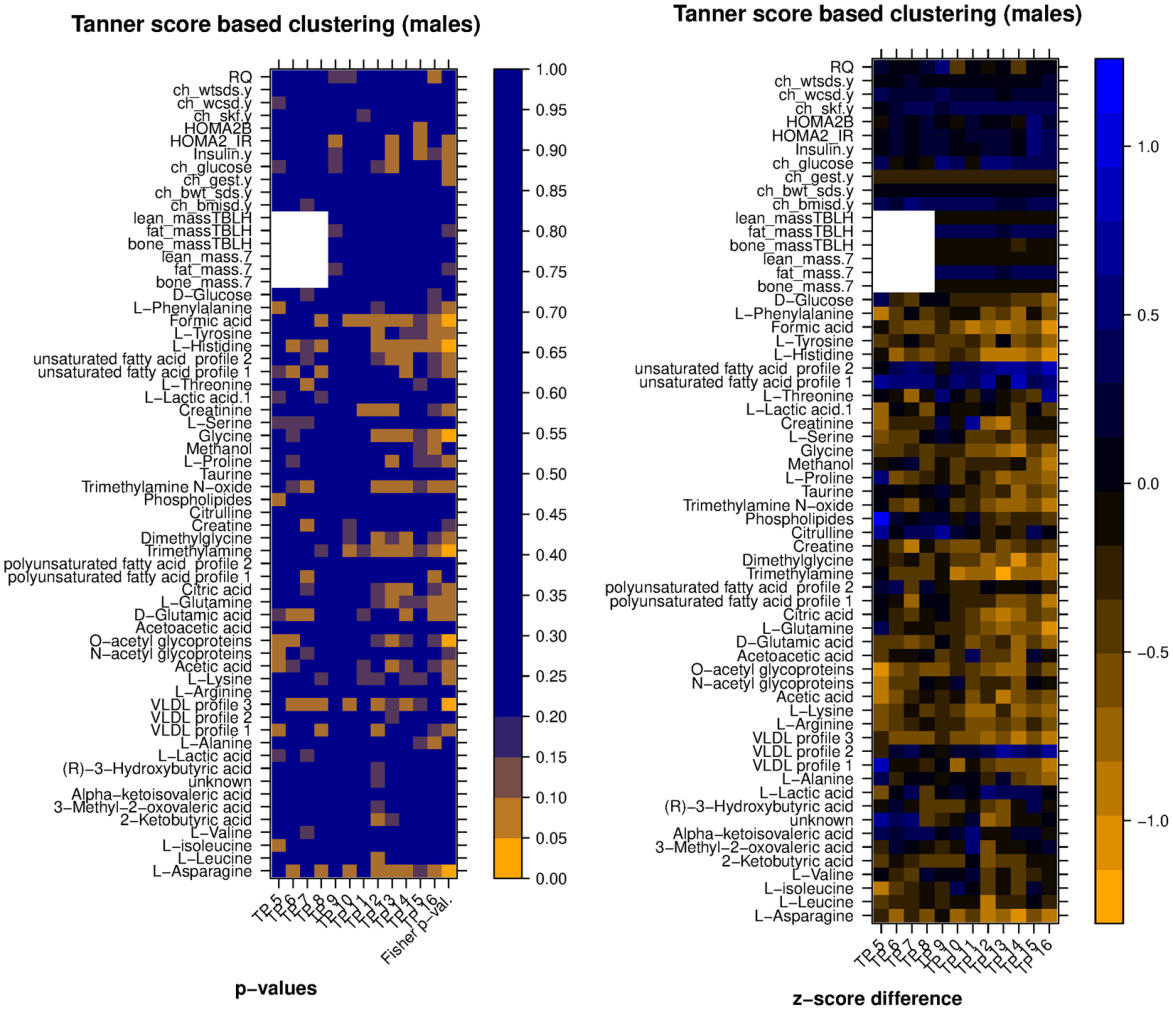
*Left:* heatmap of significance of difference between metabolic variable means between the two groups of samples (clustering by CClust according to Tanner score, all time points, males only), *Right:* heatmap of difference between the normalized averages (z-scores) of the two clusters. White boxes are fill-ins for the anthropometric variables missing time points. Legend: TennSc: Tanner score, RQ; respiratory quotient; ch_wtsds, child body weight z score; ch_wcsd, child waist circumference z score; ch_glucose, child glucose; ch_gest: child gestational age; ch_bwt_sds, child birth weight z score, TBLH, total body less head.

To select the best solutions generated by 2-way and 3-way CClust clusterings we compared the significance of the separation of average clinical variables values between the two cases. Using random permutation-based analysis, we obtained the empirical distribution of the Euclidean distance between the average curves, with one average curve computed for each cluster; we repeated the analysis for the 2-way and the 3-way clusterings, and for each one of the seven representative clinical variables. The resulting p-values unambiguously pointed to the 2-way solution as the one most distant from the random distribution of average curve separation values (Tables 4 and 5). We therefore performed the remainder of the analysis on the 2-way clustering, producing the table of random permutation p-values (Table 6), and the heatmaps of Mann-Whitney p-values (not corrected for multiple testing) and average z-score differences (Figs 10 and 11). None of the variables reached statistical significance when FDR correction was applied (Figure S7).

**Table 4:** Likelihood of observing a distance between average curves at least as large as the one obtained by CClust over n = 10000 random partitions of the subjects in the 2-way clustering of Tanner score temporal profiles.

	BMI z score	Skin fold	wcsd	Glucose	Insulin	HOMA2 IR	HOMA 2B
females	<0.001	0.02	0.01	0.29	0.01	0.02	0.02
males	0.27	0.12	0.36	0.04	0.07	0.05	0.27

An average curve for each of the n = 2 clusters was obtained for each one of the random partitions, and for each one of the seven clinical variables shown, and the Euclidean distance between curves was computed. The values in the table indicate the percentage of random partitions producing a distance at least as large as the one measured for the CClust clusters. **Legend:** wcsd: waist circumference z score.

**Table 5:** Likelihood of observing a distance between average curves at least as large as the one obtained by CClust over n = 10000 random partitions of the subjects in the 3-way clustering of Tanner score temporal profiles.

	bmisd	skf	wcsd	glucose	Insulin	HOMA2.IR	HOMA.2B
females	0.01	0.12	0.04	0.24	0.05	0.04	0.07
males	0.64	0.58	0.91	0.22	0.26	0.24	0.5

An average curve for each of the n = 3 clusters was obtained for each one of the random partitions, and for each one of the seven clinical variables shown, and the Euclidean distance between curves was computed. The values in the table indicate the percentage of random partitions producing a distance at least as large as the one measured for the CClust clusters.

**Table 6:** Empirical p-values obtained by random permutations using Tanner score as primary variable and metabolites as secondary ones.

	Female p-val	Females FDR	Males p-val	Males FDR
**(a) - Metabolites**				
L-asparagine	0.0692	0.31832	0.0111	0.12683
L-leucine	0.3119	0.56828	0.747	0.85905
L-isoleucine	0.3596	0.57881	0.7187	0.85905
L-valine	0.4512	0.6486	0.6727	0.83633
2-ketobutyric acid	0.016	0.2622	0.4674	0.65153
3-methyl-2-oxovaleric acid	0.3212	0.56828	0.7453	0.85905
Alpha-ketoisovaleric acid	0.6416	0.7027	0.933	0.933
Unassigned metabolite signal	0.8203	0.8203	0.8179	0.90449
3-hydroxybutyric acid	0.6324	0.7027	0.3558	0.56437
L-lactic acid (^1^H signal at 1.20 ppm)	0.3126	0.56828	0.5923	0.77845
L-alanine	0.1675	0.47354	0.4298	0.61784
VLDL ^1^H signal 1	0.032	0.31832	0.0869	0.23514
VLDL ^1^H signal 2	0.1514	0.47354	0.2455	0.43435
VLDL ^1^H signal 3	0.2034	0.478	0.0262	0.13391
L-arginine	0.0688	0.31832	0.3459	0.56437
L-lysine	0.0595	0.31832	0.0565	0.184
Acetic acid	0.0974	0.34465	0.0671	0.19291
N-acetyl-glycoproteins	0.2387	0.478	0.8455	0.90449
O-acetyl-glycoproteins	0.1853	0.47354	0.1084	0.27702
Acetoacetic acid	0.3649	0.57881	0.9006	0.92061
Glutamic acid	0.4827	0.64926	0.0557	0.184
Glutamine	0.6745	0.72156	0.0193	0.12683
Citric acid	0.3402	0.57881	0.06	0.184
Polyunsaturated fatty acid signal 1	0.2254	0.478	0.2706	0.46102
Polyunsaturated fatty acid signal 2	0.7596	0.79413	0.8657	0.90505
Trimethylamine	0.0117	0.2622	0.0037	0.0644
Dimethylglycine	0.0844	0.33695	0.0178	0.12683
Creatine	0.0657	0.31832	0.2041	0.4082
Citrulline	0.5608	0.69721	0.3891	0.57737
Phospholipides	0.1563	0.47354	0.6454	0.82468
Trimethylamine.N.oxide	0.7924	0.81001	0.0184	0.12683
Taurine	0.2181	0.478	0.2291	0.42909
L-proline	0.4909	0.64926	0.038	0.1748
Methanol	0.494	0.64926	0.1474	0.3231
Glycine	0.4158	0.61699	0.0427	0.17856
L-serine	0.239	0.478	0.5463	0.73911
Creatinine	0.3855	0.5911	0.0236	0.13391
L-lactic acid (^1^H signal at 4.14 ppm)	0.6255	0.7027	0.8442	0.90449
L-threonine	0.0447	0.31832	0.2332	0.42909
Unsaturated fatty acid signal 1	0.5764	0.69775	0.1326	0.32103
Unsaturated fatty acid signal 2	0.1786	0.47354	0.0579	0.184
L-histidine	0.0879	0.33695	0.0015	0.0644
L-tyrosine	0.5223	0.66738	0.1475	0.3231
Formic acid	0.0171	0.2622	0.0042	0.0644
L-phenylalanine	0.0421	0.31832	0.1997	0.4082
Glucose	0.6299	0.7027	0.3709	0.56871
**(b) - Anthropometric variables**				
Bone mass	0.0019	0.00585	0.5863	0.5688
Fat mass	0.02	0.0207	0.0948	0.5688
Lean mass	0.0027	0.00585	0.8486	0.5688
Bone mass (total body less head)	0.0031	0.00585	0.604	0.5688
Fat mass (total body less head)	0.0207	0.0207	0.0948	0.5688
Lean mass (total body less head)	0.0039	0.00585	0.8845	0.8845

Numbers shown indicate the likelihood of observing a distance between the reported metabolite average curves over n = 10000 random partitions which is as large (or larger) as the one obtained by 2-way clustering of Tanner score profiles using our algorithm. Asterisks mark the statistically significant values (p < 0.05). (a) Metabolites. (b) Anthropometric variables. Anthropometric variables were analyzed separately because they were available for a range of time points different than the other variables.

## Discussion

In this work we tackle the problem of finding relevant associations between the temporal profiles of a set of quantitative variables (clinical, anthropometric, metabolic) measured at yearly intervals between the ages of 5 and 16 for a cohort of subjects. We introduce a notion of variable prioritization by requiring the user to select a variable of particular interest (called primary variables), typically one directly associated to a phenotype under study. If the user is interested in more than on phenotype, the workflow can be repeated for each one of the associated variables. As an example, in the EarlyBird study we selected HOMA IR and Tanner score as proxies, respectively, for insulin resistance and developmental phases in childhood and puberty. The prioritization of a variable enables the partition of the subjects into groups homogeneous with respect to the temporal evolution of the related phenotype, and focuses the subsequent analysis on the discovery of associations between such phenotype and the remaining variables.

Our proposed approach solves a number of challenges that a user can expect to encounter in the implementation of this workflow. We describe a method to remedy the lack of agreement between existing clustering strategies while making use of existing results in the field. We term this method “consensus clustering”, because it is based on the identification of the two algorithms providing the largest overlap between partitions; the initial pool of algorithms comprises conceptually different approaches, in order to make the consensus non-trivial and to ensure it reflects the intrinsic structure of data.

A challenge that is relatively common in analyses encompassing a large numbers of subjects and measurements carried over several years was the non-uniform data coverage of subjects between different types of data – in other words, not all clinical, metabolic and anthropometric variables were available for every subject. The solution we used was to personalize the analysis to the range of ages and individuals available for each data type. Similarly, we decided to restrict our analysis to subjects with complete time series for the primary variables, which sacrificed some of the available data values but removed any concern about possible artifacts introduced by imputation techniques.

Another challenge was that none of the metabolic or clinical variables reached statistical significance when the subgroups were contrasted in a timepoint-wise manner, possibly due to the limited number of subjects. Interestingly, however, when we contrasted the whole time profiles of the different subgroups we obtained significant differences for several metabolic variables between HOMA IR subgroups (male subjects only), and for anthropometric variables between Tanner score subgroups (female subjects only). The uncorrected point-wise p-values reported in the heatmaps are still a valuable result, useful for prioritizing clinical variables and metabolic species in view of a possible follow-up study.

For instance, the application of our methodology to study HOMA IR trajectories in boys has revealed a limited contribution of anthropometric parameters to IR clinical behaviour. The approach also highlighted some serum metabolic patterns related to amino acid metabolism (histidine, glutamine, lysine, valine), central energy metabolism (creatine) and ketogenesis (acetate, 3D-hydroxybutyrate). Some of the metabolite patterns are consistent with previous findings, including the positive association of branch chained amino acids with IR^10^ and decreased ketogenesis in obese prepubertal children^11^. Since susceptibility to pre-diabetes and obesity later in life is influenced by various factors during childhood growth and puberty, our approach provides us with tools to explore the interactions between pubertal staging, metabolic functions and IR. One key factor currently being studied is excess of body weight during childhood which can also influence pubertal development and IR, through influences on timing of pubertal onset and pubertal hormonal levels^12^. This is exemplified here as well, through the very strong and gender-specific patterns of anthropometric and metabolites associated with pubertal staging. Such data will provide important opportunities to examine the molecular processes associated with adiposity-IR interactions during the complex period of puberty and adolescence.

In conclusion, we show that our consensus-based method is able to cluster the study subjects into groups possessing desirable properties. First, the groups are robust with respect to the method, in other words their grouping reflects the consensus among different conceptual approaches to clustering. Second, we show that the groups we obtained produced statistically significant separation between most of the main clinical variables, giving confidence that the risk groups we identified may have real biological correlates. We then proceeded to identify the metabolic, anthropometric and remaining clinical variables that correlated with the risk groups, and were able to discuss their biological relevance.

## Materials and Methods

### Study Population

The EarlyBird Diabetes Study incorporates a 1995/1996 birth cohort recruited in 2000/2001 when the children were 5 years old (307 children, 170 boys)^13^. The collection of data from the EarlyBird cohort is composed of several clinical and anthropometric variables measured on an annual basis from the age of 5 to the age of 16. Details on the measurement methods are reported in Supplementary Materials and Methods.

### Statistics

We performed all statistical calculations in the R language ver. 3.3.2^14^. Heatmaps were created in R using the RColorBrewer ver 1.1–2^15^, Lattice ver. 0.20–34^16^, GridExtra ver. 2.2.1^17^, and Grid ver. 0.7–4^14^ packages. The values in the leftmost heatmap were computed using a Matt-Whitney U test for each of the secondary variables (one per row); the test was performed between the values for subjects in the A and B groups, where A and B were the group of subjects resulting from the clustering (performed using CClust) of temporal profiles of the primary variable to which the figure refers to. The summary p-value in the last column of the heatmap was computed using the CombinePValue package^18^. The values in the rightmost heatmap were computed as follows: values for a certain variable were transformed into z-scores separately for each year (i.e. standardized to 0 mean and standard deviation of 1 considering all subjects per year), then the difference between the average z-score of group A minus the average of group B for each year was computed and represented as a color shade.

### Clustering

We used the following R packages of clustering algorithms for our analysis: MFuzz ver. 2.34.00, NBClust ver. 3.0, ssClust ver. 3.0, TSclust ver 1.2.3, Longclust ver 1.2. A comparison of the results of the application of these algorithms to the HOMA IR time profiles is reported in Fig. 4 (graphs of clustered time profiles) and in Supplementary Table ST1 (size of clusters). Additional details on the clustering methods employed in this study are reported in Supplementary Materials and Methods.

#### Measure of overlap

The measure we used for quantify the degree of overlap (consistency) between every pair of methods was the Adjusted Rand Index, the corrected for chance version of the Rand index. Though the Rand index may only assume a value between 0 and 1, the Adjusted Rand index can assume negative values if the index is less than the expected index. Given a set of n elements S and two partitions X and Y the Rand index is a fraction in which at the numerator there is the number of agreements between partitions X and Y and at the denominator the sum of number of agreements between partitions X and Y and the number of disagreements between partitions X and Y.

### Accordance

We conducted the study in accordance with the ethics guidelines of the Declaration of Helsinki II.

### Approval

Ethics approval was granted by the Plymouth Local Research Ethics Committee (1999).

### Informed consent

Parents gave written consent and children verbal assent.

### Data sharing statement

Data may be available upon request to Francois-Pierre Martin and Jonathan Pinkney, subject in particular, to ethical and privacy considerations.

## Electronic supplementary material

Supplementary Information
