# Supplementary material for: Consensus Clustering of temporal profiles for the identification of metabolic markers of pre-diabetes in childhood (EarlyBird 73)
Source: Sci Rep. 2018 Jan 23;8:1393. doi: 10.1038/s41598-017-19059-2 (PMC5780503; doi:10.1038/s41598-017-19059-2)
Supplement: Supplementary file 1 — Supplementary Information [file 41598_2017_19059_MOESM1_ESM.pdf]

Supplementary Information

Consensus Clustering of temporal profiles for the identification of metabolic markers of pre-diabetes in childhood (EarlyBird 73)

Mario Lauria[1,4,*], Maria Persico[1], Nikola Dordevic[1], Ornella Cominetti[3], Alice Matone[1], Joanne Hosking[2], Alison Jeffery[2], Jonathan Pinkney[2], Laeticia Da Silva[3], Corrado Priami[1,4,5], Ivan Montoliu[3], François-Pierre Martin[3]

[1] The Microsoft Research - University of Trento Centre for Computational and Systems Biology, Piazza Manifattura 1, 38068 Rovereto (TN), Italy

[2] Plymouth University Peninsula Schools of Medicine and Dentistry, UK

[3] Nestle Institute of Health Sciences, Lausanne, Switzerland

[4] Department of Mathematics, University of Trento, via Sommarive, 14, 38123 Povo (TN), Italy.

[5] Department of Computer Science, Stanford University, Stanford, CA

Supplementary Methods

1. Measurement of variables

1.1. Anthropometric parameters

BMI was derived from direct measurement of height (Leicester Height Measure; Child Growth Foundation, London, U.K.) and weight (Tanita Solar 1632 electronic scales), performed in blind duplicate and averaged. BMI SD scores were calculated from the British 1990 standards [1].

Physical activity was measured annually from 5 years by accelerometry (Acti-Graph [formerly MTI/CSA]) [2]. Children were asked to wear the accelerometers for 7 consecutive days at each annual time point, and only recordings that captured at least 4 days were used.

Skinfold thicknesses were measured (Holtain skinfold calipers., Holtain Ltd., Crosswell, Crymych, Dyfed, U.K.) in duplicate by one of two trained nurses over the biceps and triceps of the left arm and subscapular, suprailiac, and para-umbilical areas, and the mean was calculated for each measure in agreement with other studies [3].
From age 7y, fat mass, fat free mass and bone mass (expressed in kg) were assessed in children using the dual-energy x-ray absorptiometry (DEXA) (Lunar DPX Densitometer (GE Lunar

Corporation (Madison WI, USA)) and, owing to machine replacement, from age 9y with the Lunar Prodigy Densitometer (GE Lunar Corporation (Madison WI, USA)). Measurements did not include the head. DEXA is a precise method (CV ranges from 1-4%). The X-ray radiation dose was less than 0.01 mSv and the measurement took approximately 10 min.

Resting energy expenditure was measured by indirect calorimetry using a ventilated flow through hood technique (Gas Exchange Measurement, Nutren Technology Ltd, Manchester, UK). Performance tests reportedly show a mean error of 0.3 ± 2.0% in the measurement of oxygen consumption and 1.8 ± 1% in that of carbon dioxide production. Measurements were performed in a quiet thermoneutral room (20°C) after overnight fasting period of at least 6 hours, to minimize any effect attributable to the thermic effect of food. Data were collected for a minimum of 10 minutes and the respiratory quotient (RQ) was calculated as an indicator of basal metabolic rate (BMR).

Pubertal stage was assessed according to self-reporting of Tanner score [4]. Children are classified as pre, peri and post-pubertal stages corresponding to Tanner score 1, Tanner score 2-4 and Tanner score 5 respectively.

In addition, gestational age (weeks) and birth weight (Kg) were collected from participant medical records.

1.2. Clinical parameters

Peripheral blood was collected annually into EDTA tubes after an overnight fast and stored at -80°C. Insulin resistance (IR) was determined each year from fasting glucose (Cobas Integra 700 analyzer; Roche Diagnostics) and insulin (DPC IMMULITE) (cross-reactivity with proinsulin,1%) using the homeostasis model assessment program (HOMA-IR) [5], which has been validated in children [6].

1.3. Serum metabonomics

400 µL of blood serum were mixed with 200 µL of deuterated phosphate buffer solution 0.6 M KH_2PO_4, containing 1 mM of sodium 3-(trimethylsilyl)-[2,2,3,3-2H4]-1-propionate (TSP, chemical shift reference δH = 0.0 ppm). 550µL of the mixture were transferred into 5 mm NMR tubes.

^{1}H NMR metabolic profiles of serum samples were acquired with a Bruker Avance III 600 MHz spectrometer equipped with a 5 mm cryoprobe at 310 K (Bruker Biospin, Rheinstetten,

Germany) and processed using TOPSPIN (version 2.1, Bruker Biospin, Rheinstetten, Germany) software package as reported previously. Standard ^{1}H NMR one-dimensional pulse sequence with water suppression, Carr-Purcell-Meiboom-Gill (CPMG) spin-echo sequence with water suppression, and diffusion-edited sequence were acquired using 32 scans with 98K data-points. The spectral data (from δ 0.2 to δ 10) were imported into Matlab software with a resolution of 22K data-points (version R2013b, the Mathworks Inc, Natwick MA) and normalized to total area after solvent peak removal. Poor quality or highly diluted spectra were discarded from the subsequent analysis.

^{1}H-NMR spectrum of human blood plasma enables the monitoring of signals related to lipoprotein bound fatty acyl groups found in triglycerides, phospholipids and cholesteryl esters, together with peaks from the glyceryl moiety of triglycerides and the choline head group of phosphatidylcholine. This data also covers quantitative profiling of major low molecular weight molecules present in blood. Based on internal database, representative signals of metabolites assignable on ^{1}H CPMG NMR spectra were integrated, including asparagine, leucine, isoleucine, valine, 2-ketobutyric acid, 3-methyl-2-oxovaleric acid, alpha-ketoisovaleric acid, (R)-3-hydroxybutyric acid, lactic acid, alanine, arginine, lysine, acetic acid, N-acetyl glycoproteins, O-acetyl glycoproteins, acetoacetic acid, glutamic acid, glutamine, citric acid, dimethylglycine, creatine, citrulline, trimethylamine, trimethylamine N-oxide, taurine, proline, methanol, glycine, serine, creatinine, histidine, tyrosine, formic acid, phenylalanine, threonine, and glucose. In addition, in diffusion edited spectra, signals associated to different lipid classes were integrated, including phospholipids containing choline, VLDL subclasses, unsaturated and polyunsaturated fatty acid. The signals are expressed in arbitrary unit corresponding to a peak area normalized to total metabolic profiles, which is representative of relative change in metabolite concentration in the serum.

We note that complete temporal series were available only for 26 subjects across the 5-16 year time points. However since metabolic data time was analyzed per time point, we were not restricted to subjects with complete series.

2. Clustering algorithms

2.1. Mfuzz package

The R package Mfuzz was developed in the context of microarrays analysis to overcome shortcomings of conventional hard clustering techniques such as sensitivity to noise and information loss [7]. In contrast, soft clustering methods can assign a gene to several clusters. Soft clustering has been implemented using the fuzzy c-means algorithm; it is based on the iterative optimization of an objective function to minimize the variation of objects within clusters. Poorly clustered objects have decreased influence on the resulting clusters making the clustering process less sensitive to noise. As a result, fuzzy c-means produces gradual membership values MUij of item i between 0 and 1 indicating the degree of membership of this item for cluster j. The number of clusters and the so-called fuzzification parameter m have to be chosen. By variation of both parameters, users can probe the stability of obtained clusters as well as the global clustering structure. For the fuzzy logic based classifications, the fuzzifier was specified in the caption of the figures.

2.2. NbClust package

The R package NbClust is essentially aimed at cluster validation and finding optimal number of clusters [8]. The package allows an easy comparison of clustering schemes and establishing of optimal number of clusters according to the majority rule. The schemes used for obtaining this result have abbreviation nbclust1 and nbclust3 and can be described respectively as 1) a k-means clustering using euclidean distance evaluated using all indices implemented in the package and 2) a hierarchical clustering refined using the agglomerative strategy named Ward.D, using a dissimilarity matrix calculated with euclidean distances evaluated using all indices implemented in the package. The parameters related to minimal and maximal number of clusters to evaluate were set respectively to min.nc=2 and max.nc=4.

2.3. ssClust package

The R package SSCLUST implements statistical methods for clustering time-series gene expression data [9] . In particular, Smoothing Spline Clustering is useful for clustering genes in microarray experiments performed over several time-points, for example, over the course of development, a drug treatment, or other temporally based experiments. It allows direct discovery of related patterns of gene expression and their underlying functions (curves) from data without a priori specification of either cluster number or functional form. Smoothing spline clustering (SSC) models natural properties of gene expression over time, taking into account natural differences in gene expression within a cluster of similarly expressed genes, the effects of experimental measurement error, and

missing data. Furthermore, SSC provides a visual summary of each cluster 4s gene expression function and goodness-of-fit by way of a mean curve construct and its associated confidence bands.

2.4. TSclust package

The R package TSclust contains a set of measures of dissimilarity between time series to perform time series clustering [10]. Metrics based on raw data, on generating models and on the forecast behavior are implemented. It is aimed to implement a large set of well-established peer-reviewed time series dissimilarity measures, including measures based on raw data, extracted features, underlying parametric models, complexity levels, and forecast behaviors. Computation of these measures allows the user to perform clustering by using conventional clustering algorithms, i.e. hierarchical clustering (called tscluCORThc in the following) and partitional clustering (tscluCORTpam in the following); additional utilities related to time series clustering are also provided, such as cluster evaluation metrics. For calculating the dissimilarity matrix, the core step in the TSclust workflow, we have used the distance measure named CORT: it computes an adaptive dissimilarity index between two time series that covers both dissimilarity on raw values and dissimilarity on temporal correlation behaviors.

2.5. Longclust package

The R package longclust implements a Model-Based Clustering and Classification strategy tailored for Longitudinal Data [11,12]. Clustering or classification of longitudinal data are based on a mixture of multivariate t or Gaussian distributions with a Cholesky-decomposed covariance structure.

References.

1. Cole, T. J., Freeman, J. V & Preece, M. A. Body mass index reference curves for the UK, 1990. *Arch. Dis. ChildhoodArch Dis Child* **73,** 25–29 (1995).

2. Puyau, M. R., Adolph, A. L., Vohra, F. a & Butte, N. F. Validation and calibration of physical activity monitors in children. *Obes. Res.* **10,** 150–157 (2002).

3. Lohman, T. G., Roche, A. F., Martorell, R. & Champaign, I. H. K. B. *Anthropometric standardization reference manual*. (Champaign, IL: Human Kinetics Books, 1988).

4. Tanner, J. M. & Whitehouse, R. H. Clinical longitudinal standards for height, weight, height velocity, weight velocity, and stages of puberty. *Arch Dis Child* **51,** 170–179 (1976).

5. Matthews DR, Hosker JP, Rudenski AS, Naylor BA, Treacher DF, T. R. Homeostasis model assessment insulin resistance and beta-cell function from fasting plasma glucose and insulin concentrations in man. *Diabetologia* **28,** 412–9 (1985).

6. Gungor, N., Saad, R., Janosky, J. & Arslanian, S. Validation of surrogate estimates of insulin sensitivity and insulin secretion in children and adolescents. *J. Pediatr.* **144,** 47–55 (2004).

7. Kumar, L. & E Futschik, M. Mfuzz: a software package for soft clustering of microarray data. *Bioinformation* **2,** 5–7 (2007).

8. Charrad, M., Ghazzali, N., Boiteau, V. & Niknafs, A. NbClust : An R Package for Determining the Relevant Number of Clusters in a Data Set. *J. Stat. Softw.* **61,** 1–36 (2014).

9. Ma, P., Castillo-Davis, C. I., Zhong, W. & Liu, J. S. A data-driven clustering method for time course gene expression data. *Nucleic Acids Res.* **34,** 1261–1269 (2006).

10. Montero, P. & Vilar, J. TSclust: An R Package for Time Series Clustering. *JSS J. Stat. Softw.* **62,** 1–43 (2014).

11. McNicholas, P. D. & Murphy, T. B. Model-based clustering of longitudinal data. *Can. J. Stat.* **38,** 153–168 (2010).

12. McNicholas, P. D. & Subedi, S. Clustering gene expression time course data using mixtures of multivariate t-distributions. *J. Stat. Plan. Inference* **142,** 1114–1127 (2012).

Supplementary tables and figures

Table ST1. Comparison of size of clusters identified for the same set of HOMA IR temporal profiles by the different tools. *(a)* Early time points. *(b)* Late time points. Some tools allow the user to choose the number of clusters to produce: in this case the tool was configured to produce two clusters. For the tools for which there was no such option and that produced more than two clusters, the two largest ones were selected (cluster id shown in parenthesis). The A, B designation was based on size, with A denoting the largest cluster.

(a)

	Males		Females	
	Group A	Group B	Group A	Group B
Mfuzz	23	11	4	4
kmInMfuzz	25	9	5	3
SSClust	25	9	5	3
nbclust1	31	3	NA	NA
nbclust3	12 (cl #3)	10 (cl #4)	NA	NA
tscluCORThc	27	7	4	4
tscCORTpam	22	12	5	3
loclu	12 (cl #1)	11 (cl #2)	8	0

(b)

	Males		Females	
	Group A	Group B	Group A	Group B
Mfuzz	31	25	10	6
kmInMfuzz	34	22	11	5
SSClust	34	22	11	5
nbclust1	39	17	8 (cl #3)	7 (cl #2)
nbclust3	26 (cl #3)	17 (cl #2)	12	4
tscluCORThc	46	10	9	7
tscCORTpam	34	22	8	8
loclu	22 (cl #3)	10 (cl #4)	10	6

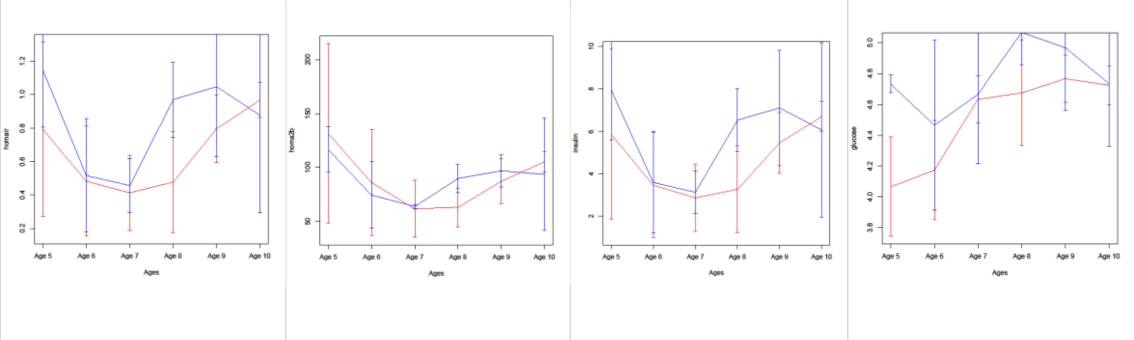

(a) (b) (c) (d)

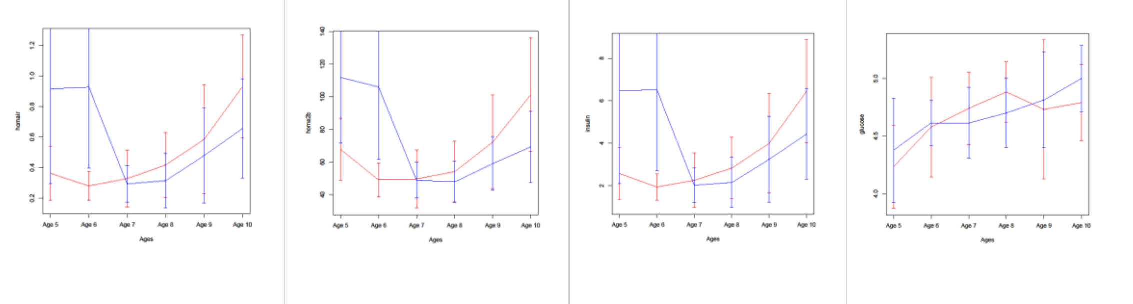

(e) (f) (g) (h)

Figure S0. Average trajectories computed for the A, B clusters of Clinical variable HOMAIR temporal profiles (early time points). Top: female subjects (n=10 and n=5 for group A, B respectively). Bottom: male subjects subjectss (n=31 and n=22 for group A, B respectively). Average curves for clinical variables HOMAIR (a, e), HOMA2B (b, f), Insulin (c, g), Glucose (d, h) with standard deviation values represented as error bars. Average curves corresponding to consensus cluster A are in red, the ones from consensus cluster B are in blue.

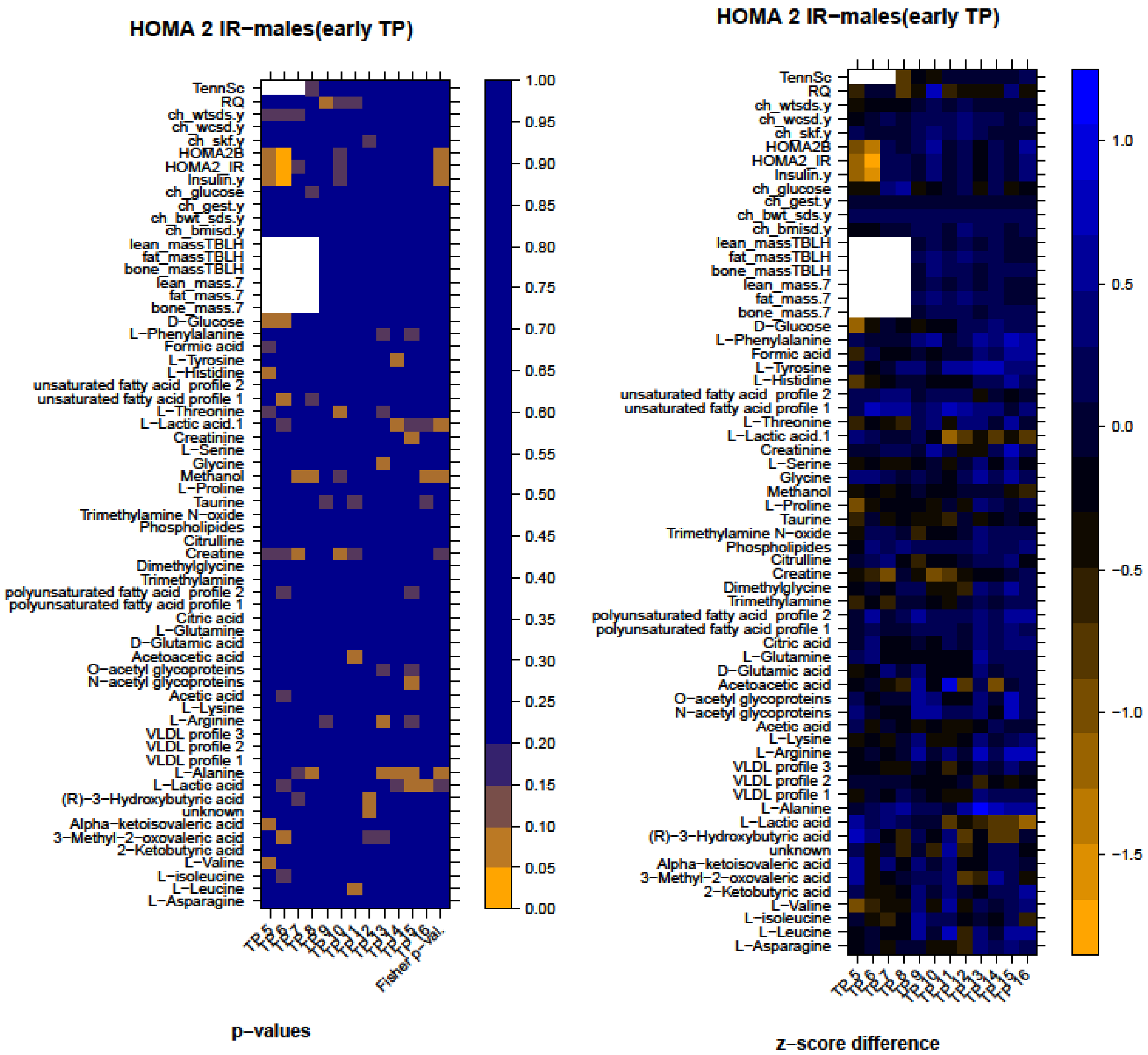

Figure S1. *Left:* heatmap of significance of difference between metabolic/clinic variable means between the two groups of samples (clustering according to HOMA IR, early time points, males only), *Right:* heatmap of difference between the normalized averages (z-scores) of the two clusters.

Legend: TennSc : Tanner score, RQ; respiratory quotient; ch_wtsds, child body weight z score; ch_wcsd, child waist circumference z score; ch_glucose, child glucose; ch_gest: child gestational age; ch_bwt_sds, child birth weight z score, TBLH, total body less head;

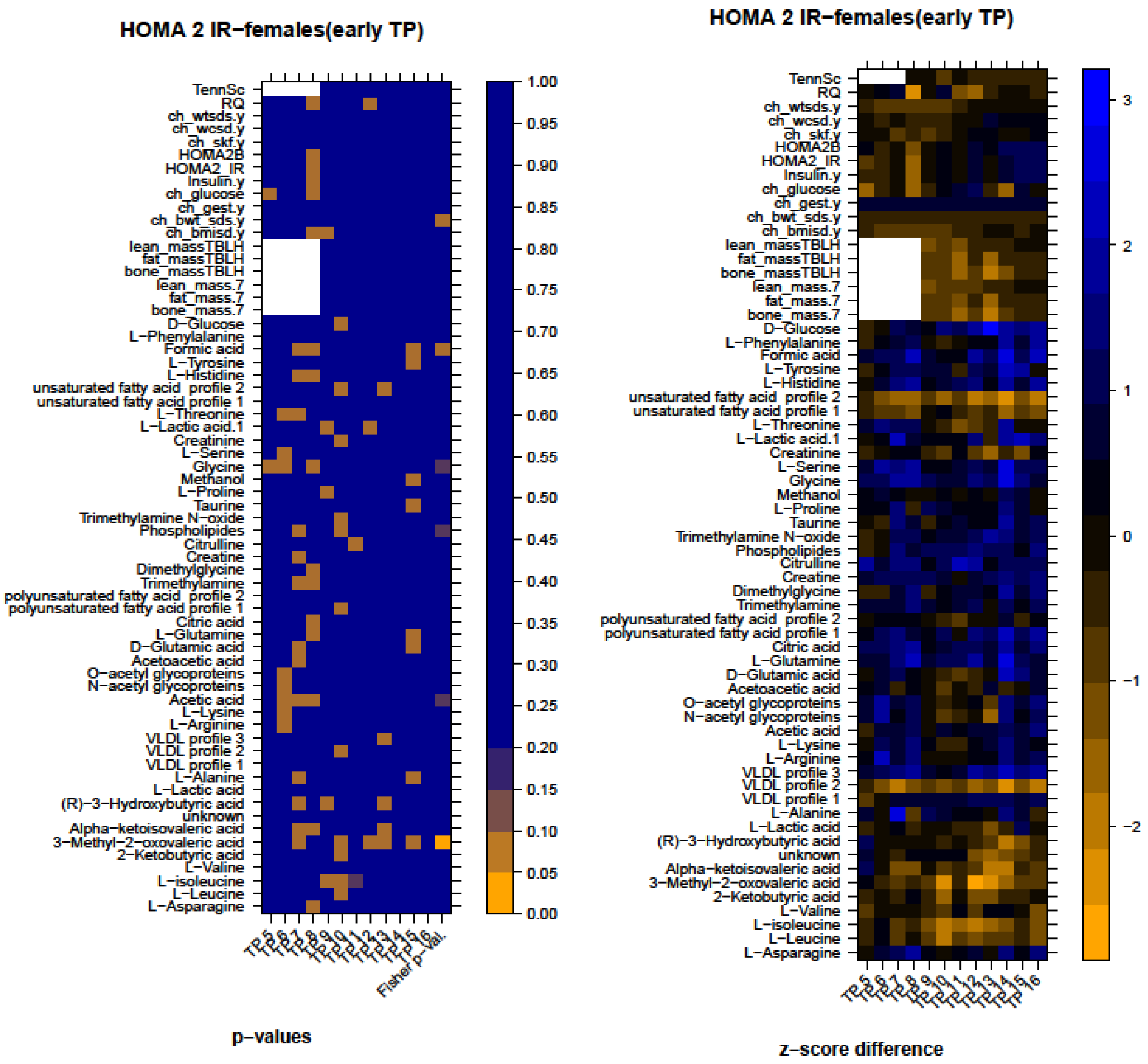

Figure S2. *Left:* heatmap of significance of difference between metabolic/clinic variable means between the two groups of samples (clustering according to HOMA IR, early time points, females only), *Right:* heatmap of difference between the normalized averages (z-scores) of the two clusters.

Legend: TennSc : Tanner score, RQ; respiratory quotient; ch_wtsds, child body weight z score; ch_wcsd, child waist circumference z score; ch_glucose, child glucose; ch_gest: child gestational age; ch_bwt_sds, child birth weight z score, TBLH, total body less head;

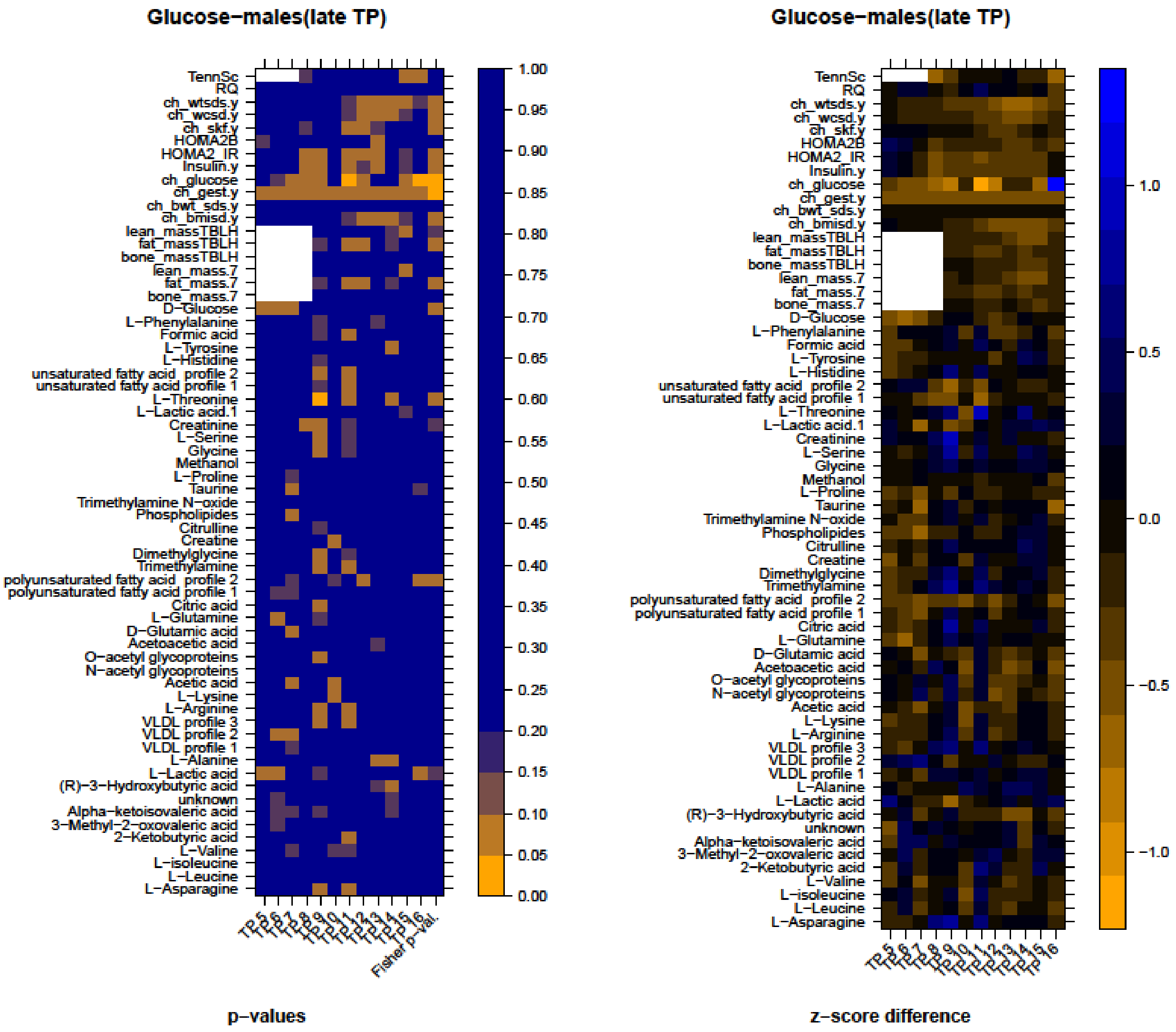

Figure S3. *Left:* heatmap of significance of difference between metabolic/clinic variable means between the two groups of samples (clustering according to Glucose, late time points, males only), *Right:* heatmap of difference between the normalized averages (z-scores) of the two clusters.

Legend: TennSc : Tanner score, RQ; respiratory quotient; ch_wtsds, child body weight z score; ch_wcsd, child waist circumference z score; ch_glucose, child glucose; ch_gest: child gestational age; ch_bwt_sds, child birth weight z score, TBLH, total body less head;

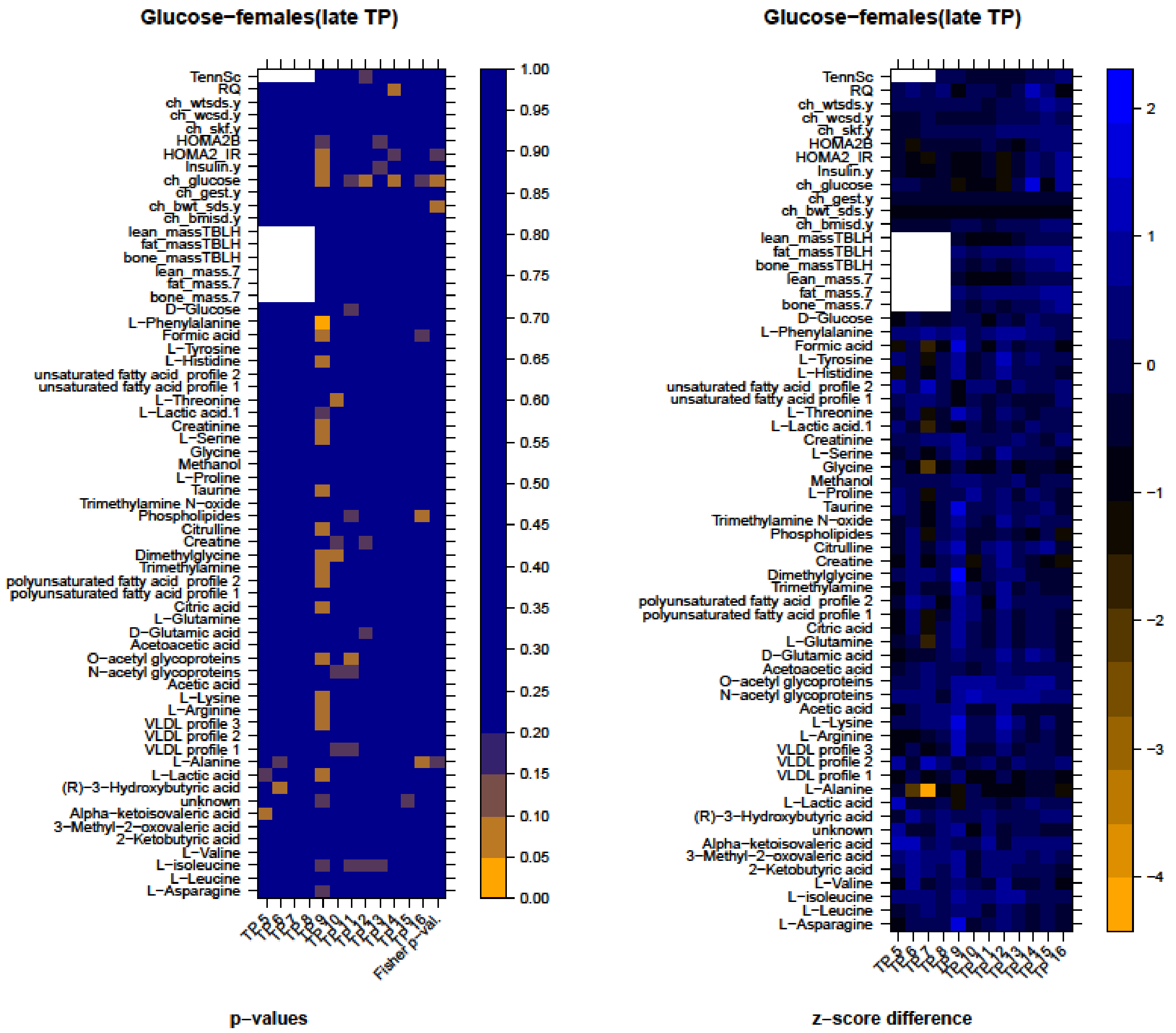

Figure S4. *Left:* heatmap of significance of difference between metabolic/clinic variable means between the two groups of samples (clustering according to Glucose, late time points, females only), *Right:* heatmap of difference between the normalized averages (z-scores) of the two clusters.

Legend: TennSc : Tanner score, RQ; respiratory quotient; ch_wtsds, child body weight z score; ch_wcsd, child waist circumference z score; ch_glucose, child glucose; ch_gest: child gestational age; ch_bwt_sds, child birth weight z score, TBLH, total body less head;

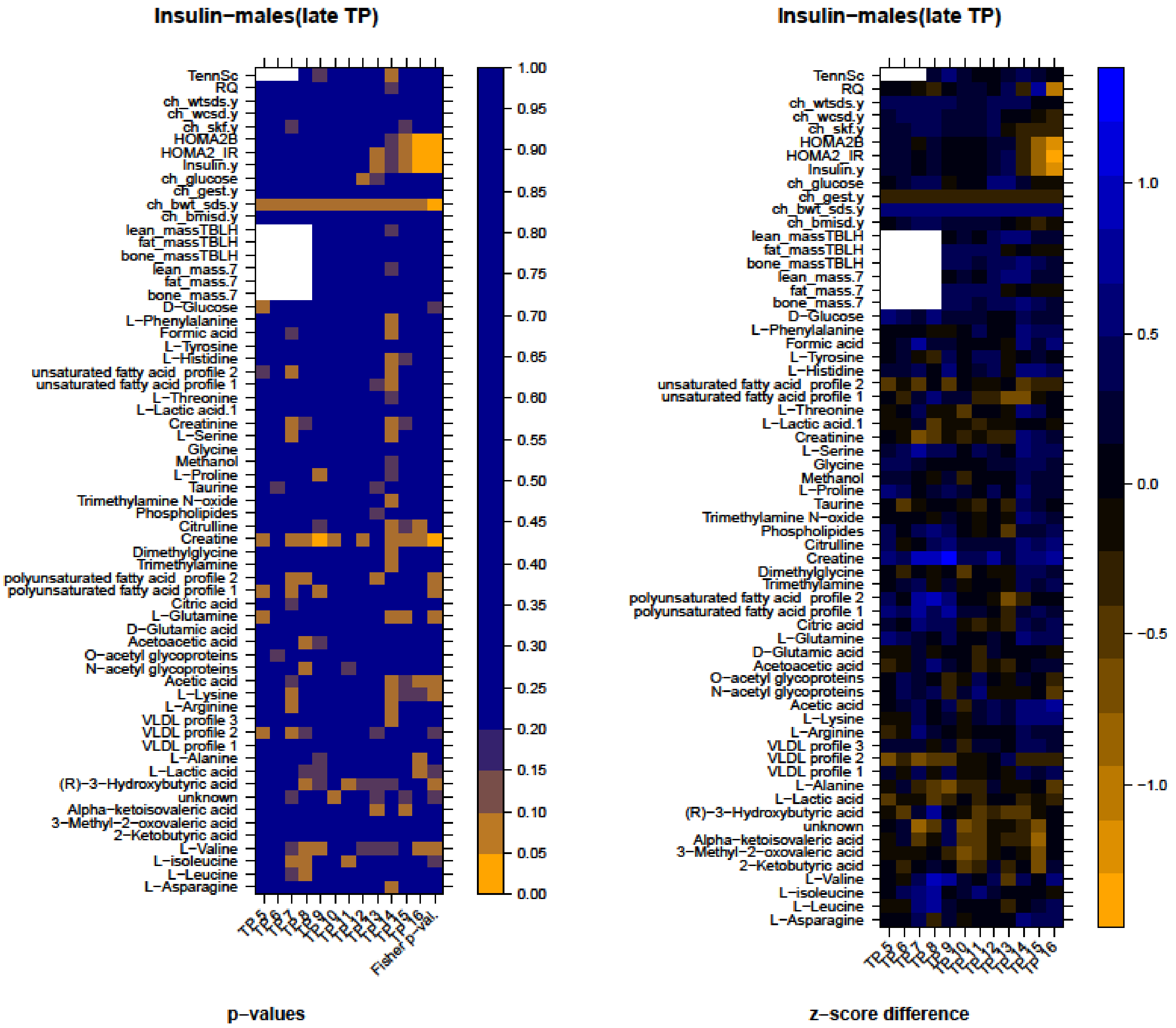

Figure S5. *Left:* heatmap of significance of difference between metabolic/clinic variable means between the two groups of samples (clustering according to Insulin, late time points, males only), *Right:* heatmap of difference between the normalized averages (z-scores) of the two clusters.

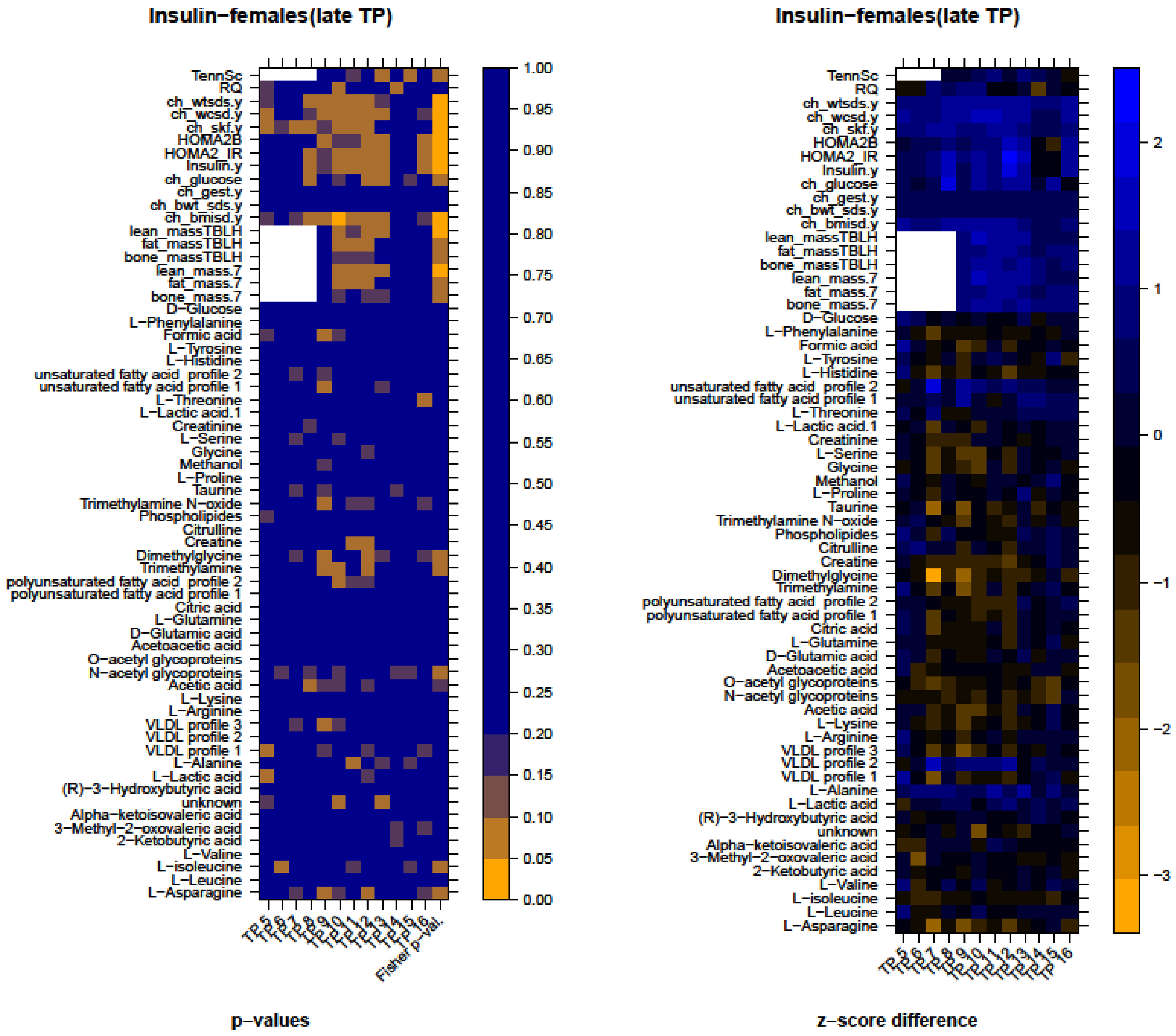

Figure S6. *Left:* heatmap of significance of difference between metabolic/clinic variable means between the two groups of samples (clustering according to Insulin, late time points, females only), *Right:* heatmap of difference between the normalized averages (z-scores) of the two clusters.

Legend: TennSc : Tanner score, RQ; respiratory quotient; ch_wtsds, child body weight z score; ch_wcsd, child waist circumference z score; ch_glucose, child glucose; ch_gest: child gestational age; ch_bwt_sds, child birth weight z score, TBLH, total body less head;

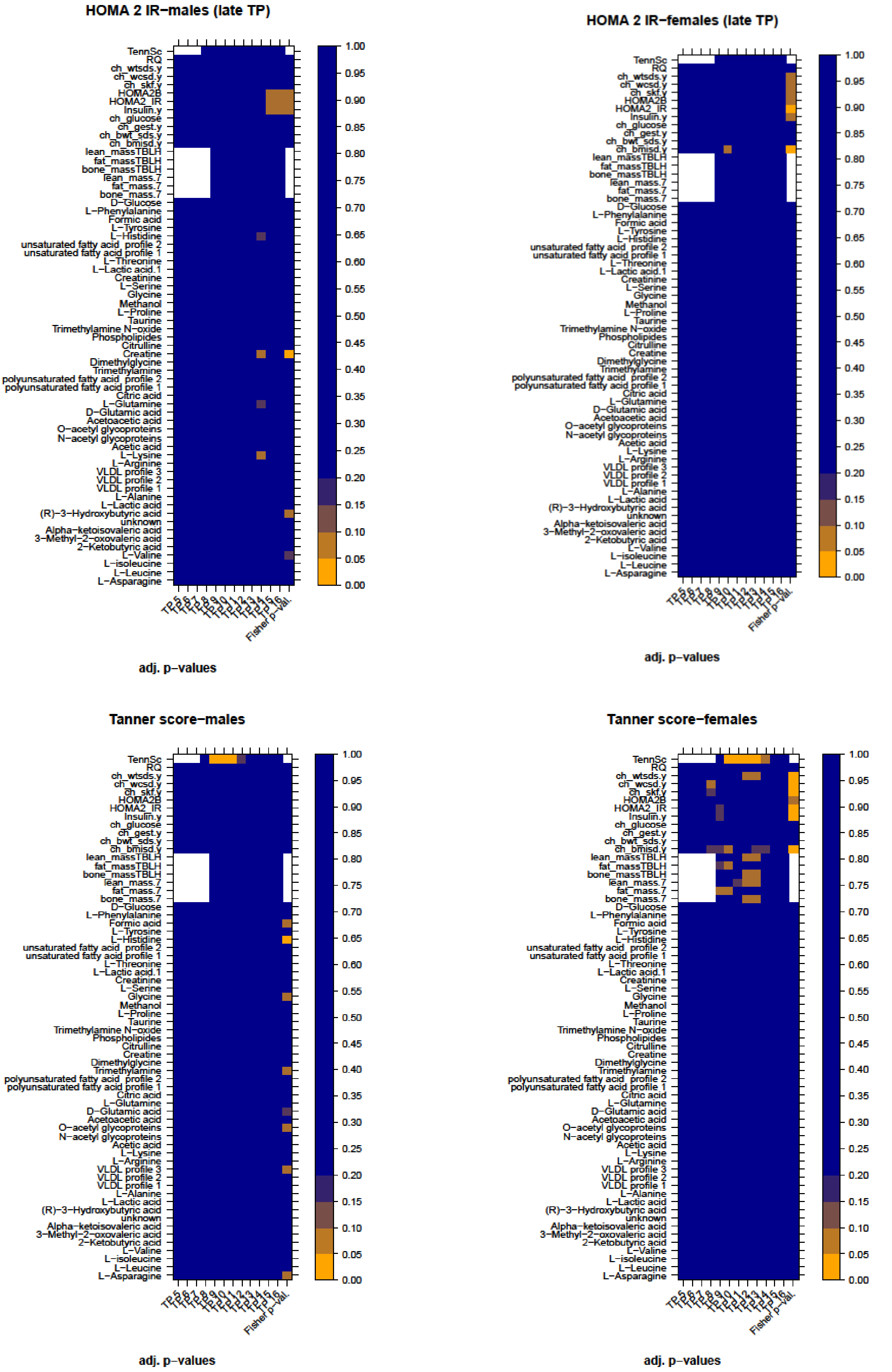

Figure S7. Heatmaps based on corrected p-values.

Legend: TennSc : Tanner score, RQ; respiratory quotient; ch_wtsds, child body weight z score; ch_wcsd, child waist circumference z score; ch_glucose, child glucose; ch_gest: child gestational age; ch_bwt_sds, child birth weight z score, TBLH, total body less head;

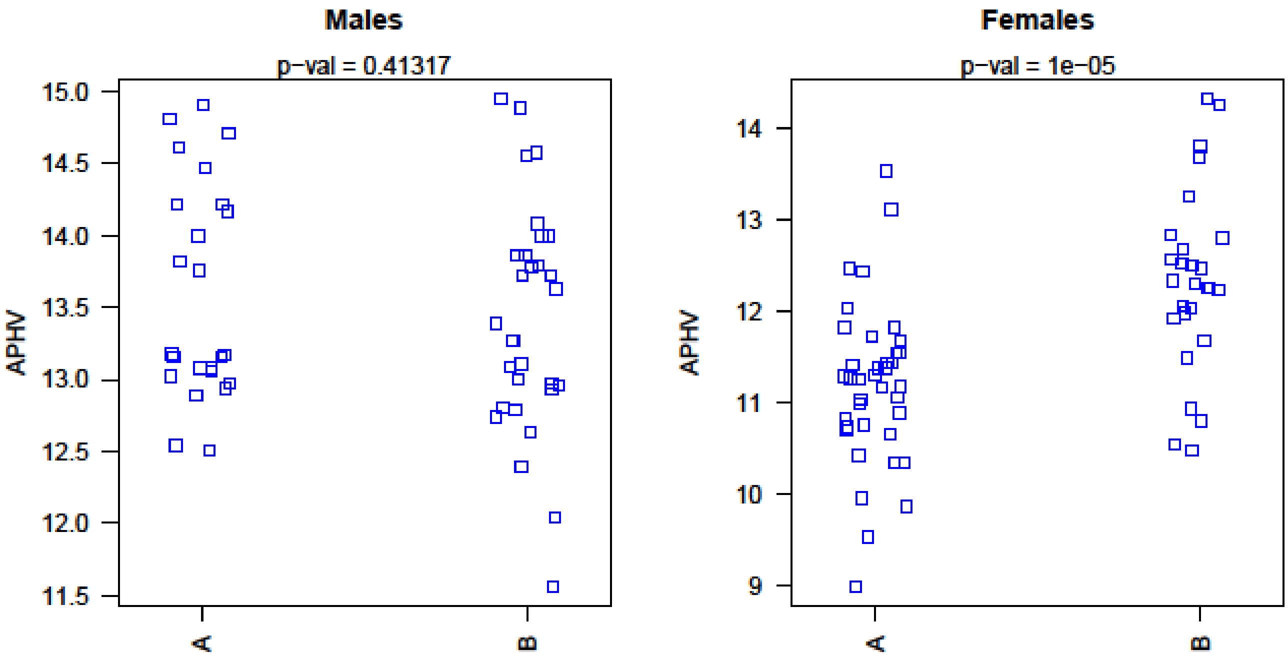

Figure S8. Distribution of values for the Age of Peak Height Velocity (APHV) clinical variable in the A and B groups obtained by Tanner score clustering of subjects. *Left:* male subjects. *Right:* female subjects.

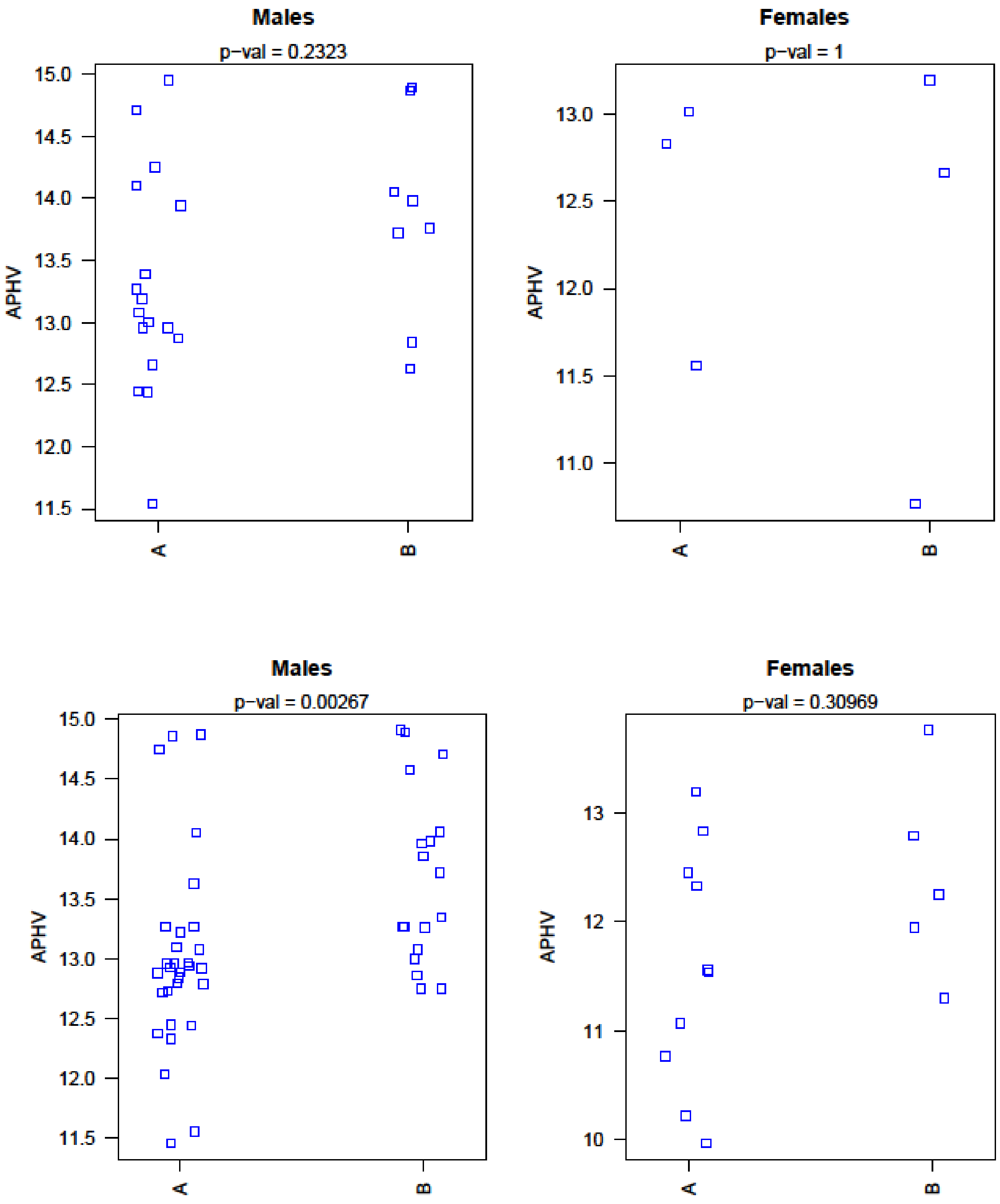

Figure S9. Distribution of values for the Age of Peak Height Velocity (APHV) clinical variable in the A and B groups obtained by HOMA-IR clustering of subjects. Subject groups A and B were obtained by clustering HOMA IR time profiles separately for early (ages 5-10) and for late (ages 11-16) time points. Top: early time points. Bottom: late time points.
